# Change in physical activity from adolescence to early adulthood: a systematic review and meta-analysis of longitudinal cohort studies

**DOI:** 10.1136/bjsports-2016-097330

**Published:** 2017-07-24

**Authors:** Kirsten Corder, Eleanor Winpenny, Rebecca Love, Helen Elizabeth Brown, Martin White, Esther van Sluijs

**Affiliations:** UKCRC Centre for Diet and Activity Research (CEDAR) at the MRC Epidemiology Unit, University of Cambridge School of Clinical Medicine, Institute of Metabolic Science, Cambridge Biomedical Campus, Cambridge, UK

**Keywords:** Physical Activity, Adolescent

## Abstract

**Objective:**

To systematically review and meta-analyse how physical activity (PA) changes from adolescence to early adulthood (13–30 years).

**Data sources:**

Seven electronic databases were searched: Medline, Embase, PsycInfo, SCOPUS, ASSIA, SPORTdiscus and Web of Science.

**Eligibility criteria for selecting studies:**

English-language, longitudinal studies (from 01/1980 to 01/2017) assessing PA ≥twice, with the mean age of ≥1 measurement in adolescence (13–19 years) and ≥1 in young adulthood (16–30 years) were included. Where possible, data were converted to moderate-to-vigorous physical activity (MVPA) min/day, and meta-analyses were conducted between weighted mean differences (WMDs) in adolescence and adulthood. Heterogeneity was explored using meta-regression.

**Results:**

Of 67 included studies, 49 were eligible for meta-analysis. PA was lower during adulthood than adolescence WMD (95% CI) −5.2 (−7.3 to –3.1) min/day MVPA over mean (SD) 3.4 (2.6) years; heterogeneity was high (I^2^ >99.0%), and no predictors explained this variation (all p>0.05). When we restricted analysis to studies with data for males (n=29) and females (n=30) separately, there were slightly larger declines in WMD (−6.5 (−10.6 to –2.3) and −5.5 (−8.4 to −2.6) min/day MVPA) (both I^2^ >99.0%). For studies with accelerometer data (n=9), the decline was −7.4 (−11.6 to –3.1) and longer follow-up indicated more of a decline in WMD (95% CI) (−1.9 (−3.6 to –0.2) min/day MVPA), explaining 27.0% of between-study variation. Of 18 studies not eligible for meta-analysis, nine statistically tested change over time: seven showed a decline and two showed no change.

**Conclusion:**

PA declines modestly between adolescence and young adulthood. More objective longitudinal PA data (eg, accelerometry) over this transition would be valuable, as would investigating how PA change is associated with contemporaneous social transitions to better inform PA promotion interventions.

**Registration:**

PROSPERO ref:CRD42015030114.

## Introduction

Physical activity in young people has been associated with a reduced risk of obesity,^1^ metabolic syndrome,^2^ beneficial effects on mental health,^3^ school performance,^4^ sleep duration^5^ and well-being.^6^ Most evidence suggests that physical activity declines with age throughout adolescence, although evidence on the magnitude of the decline is equivocal.^7^ In addition, inactivity may track into adulthood,^8^ resulting in greater health risks later in life.^9^

The transition to adulthood often includes major life changes including relocation, changes in living arrangements, academic and employment status and new social roles.^10^ Such changes likely influence health-related behaviours.^11^ Evidence suggests that the transition from adolescence to early adulthood,^12^ including from high school to post high school,^13^ might particularly influence physical activity declines. However, the limited evidence is dominated by self-reported behaviour, which is susceptible to various forms of bias and there appears to be little objective data available.

How physical activity changes over the transition from adolescence to adulthood is unclear. Therefore, we asked, ‘How does physical activity change during the period from adolescence to early adulthood (age 13 to 30)?’ To our knowledge, no reviews have investigated physical activity over this transition, but the synthesis of the available evidence via a systematic review is important to better characterise change in physical activity over this important time.

Identifying characteristics of any changes may highlight potential strategies for physical activity promotion, including potentially distinguishing the respective importance of adolescence and young adulthood as targets for physical activity promotion. We aimed to identify the state of the evidence and pinpoint knowledge gaps regarding changes in physical activity occurring during the transition to adulthood. Specifically, we assessed whether physical activity changes between adolescence (13–19 years) and early adulthood (16–30 years) and the extent of this change; we hypothesised that physical activity declines over the transition from adolescence to adulthood.

## Methods

We conducted a literature search of longitudinal observational studies providing data on physical activity in young people (PROSPERO ref: CRD42015030114). We searched seven electronic databases (Medline via PubMed, Embase via Ovid, PsycInfo (EBSCO), SCOPUS, ASSIA via ProQuest, SPORTdiscus and Web of Science Core Collection) from 1980 to January 2017. We only included data collected after 1980 to ensure that results are relevant to contemporary populations while still representing 36 years of research. The search strategy focused on three themes: participants (including adolescents and young adults), outcomes (including moderate-to-vigorous physical activity (MVPA) and physical activity) and study type (including longitudinal and prospective). The full list of terms is available in online supplementary table 1; the search was conducted by EW (research associate). The search was undertaken in parallel with a search on dietary behaviour so the search strategy also contains diet-related terms.

10.1136/bjsports-2016-097330.supp1 Supplementary file 1

### Inclusion criteria

Inclusion was restricted to published longitudinal data with at least two data collection points within the age range; the mean age of at least one measurement was required to be in adolescence (13–19 year) and at least one in young adulthood (16–30 years). The overlapping age definitions enable inclusive definitions of both periods and avoid arbitrary cut-offs limiting our search results. Thirty years old has been suggested as the end of young adulthood,^14^ and adolescence is defined by the WHO as 10–19 years.^15^ Transitions from childhood into adolescence or throughout adolescence have been covered by previous reviews,^7 16^ so we begin our review at age 13 to capture transitions occurring from mid-adolescence to early adulthood. The physical activity measurements included in the papers had to be at least 1 year apart. We were interested in a wide variety of absolute measures of physical activity but excluded studies presenting only sedentary behaviour. Studies reporting no absolute physical activity over at least two time-points (including some tracking studies or those only reporting % participants meeting guidelines) were excluded. We did not approach authors for additional information as we did not want to bias this review in the event of differential author response. Contacting authors may help to ameliorate incomplete reporting of outcomes that may lead to potential bias in systematic reviews of trials^17^; this is less of an issue in observational data.^18^ Moreover, previous reviews report limited response (42%) after multiple requests, with the data received biased towards more recent studies.^19^ All articles published in the English language in a scientific journal regardless of country of origin and containing data collected 1980 onwards were considered for inclusion. Inclusion criteria are presented in table 1.

**Table 1:** Inclusion criteria

	Inclusion criteria	Exclusion criteria
Setting	All countries	None
Participants	Those aged between 13 and 30 years Mean age of at least one measurement in adolescence (13–19 years old) and one in adulthood (16–30 years old)	Those aged below 13 years of age or above 30 years of age ≥2 measurements <16 years old or ≥2 measurements >19 years old Participant groups selected based on a pre-existing health condition (including obesity and eating disorders)
Outcomes	At least one measure of physical activity, for example: MVPA Total activity Sport participation Energy expenditure	Studies including no PA outcomes Studies reporting tracking of PA behaviours only with no data on absolute change in behaviour Studies reporting solely on sedentary behaviour
Study type	Longitudinal quantitative studies, with data collection including on specified outcomes over ≥2 time-points (minimum 1 year apart) where mean age of the cohort is between ages 13 and 30 (with at least one measurement in adolescence (13–19 years old) and one in adulthood (16–30 years old)	Other quantitative study types Qualitative studies Interventions Trial analyses
Publication type	Journal article	Conference abstract, study protocol, report, dissertation, book and professional journal
Publication year	1980 onwards	Before 1980
Language	English	All other languages

MVPA, moderate-to-vigorous physical activity; PA, physical activity.

Three reviewers (KC/EW/EvS) independently reviewed the same 1500 results from the initial title and abstract search (in 3 sets of 500). Iterative comparison and discussion allowed for development of a consistent screening approach; screening of all remaining titles/abstracts was divided between KC, EW and EvS. Subsequently, two reviewers (KC/RL) independently screened full texts for inclusion and discussed discrepancies to reach consensus. Hand searching of the references identified three additional full texts, none of which were included.

Where data from the same study was reported in multiple papers, the following decision hierarchy was applied: (1) the paper with most time-points; (2) additional papers with further time-points; (3) objective data (vs self-report); (4) largest number of baseline participants; (5) earliest publication date; and (6) largest number of follow-up participants.

### Data extraction

KC conducted data extraction with 100% checked for accuracy (RL); discrepancies were resolved by discussion. Data extracted included: baseline date (date of the first wave meeting the inclusion criteria), study name, country, ethnicity, sex, socioeconomic status, and number of participants, age, physical activity measurement method and physical activity data at each relevant time-point. Any analysis of change was also recorded including whether any adjustments were made. Data were extracted for males and females separately where possible; if not available, data for the whole sample were extracted, and data for other subgroups were extracted if an overall measure by sex was not available.^20–28^ If data were not presented by sex or overall, data summarised by ethnicity were extracted if available^22 23 26 28^ and otherwise for whichever variable resulted in the least subgroups.

This review originally intended to address a second research question: ‘How do factors such as socio-economic status or particular lifestyle transitions influence these changes in physical activity behaviour?’ Data extraction revealed there was little heterogeneous information available to address this question, and we therefore only focus on the main research question.

### Assessment of risk of bias

Risk of bias was scored using an adapted version of a previously used scale (Effective Public Health Practice Project Quality Assessment Tool), which has been explained in detail previously^29^ and used in other physical activity reviews.^30^ Participant representativeness, study size, participant drop-out, quality of physical activity data and quality of change analyses were rated. These criteria were assessed by two independent reviewers (KC and HEB/EvS); in case of disagreement, consensus was derived by discussion with a further reviewer (EW). Each item was scored as ‘Strong’, ‘Moderate’ or ‘Weak’; if a paper provided insufficient information, then it was scored as ‘Weak’. Scores for each item were summed, and quality was defined as ‘Weak’ when at least one item was classed as ‘Weak’. Papers were classed as ‘Strong’ when three out of the five criteria were rated as ‘Strong’, and no items were scored as ‘Weak’; other studies were classed as ‘Moderate’. We scored the size of a study as ‘Strong’ if there were over 1000 participants at the follow-up with the least participants; using 1000 as the threshold was an arbitrary decision, but we deemed this suitable considering 22% of the included studies had ≥1000 participants. Analyses were scored as ‘Strong’ if they used a suitable statistical method to assess change (such as regression) and included appropriate adjustment for potential confounders. Studies were scored as ‘Strong’ if objective methods were used to assess physical activity and were the same at all time-points. If validity and reliability of self-reported methods were stated, and were the same over both time-points, then they were rated as ‘Moderate’. Scoring details are summarised in online supplementary table 2.

### Data preparation

Wherever possible, values from all included studies were converted to a common metric to enable meta-analysis of physical activity change. The metric chosen was the original unit reported for all studies with accelerometer data (MVPA min/day). If a paper presented more than one physical activity outcome, the one most comparable with MVPA was selected and otherwise the value most easily converted to MVPA min/day. The extent of data conversion was recorded for each study so it could be included in meta-regression. Conversion was defined as minimal when reported data were in min/day, min/week or similar and as extensive when any other conversion was necessary. As in a previous review,^31^ pedometer counts were translated to approximate MVPA using 100 steps/min, and a bout of activity was classed as 30 min. For data reporting MET minutes or MET times/week, the value reported was divided by the appropriate MET value if stated in the paper (or five for MVPA to account for the whole range of MVPA (≥4 METS^32^)).

Due to the overlapping adolescent and adulthood time periods, decisions were made to class study time-points as ‘adolescence’ or ‘adulthood’. If only two time-points per study were available, the first was classed as adolescence and the second as adulthood. For studies with >2 time-points, at least one time-point was included as adolescence and at least one as adulthood, any further time-points were divided at 17.5 years old (the midpoint between the adolescent and adulthood age ranges). The additional time-points <17.5 years were incorporated with the adolescent estimate and time-points ≥17.5 years were incorporated with the adult estimate so that mean activity values could be used where necessary; each time-point was used once. Sample size at follow-up(s) was assumed to be that of baseline when time-point specific participant numbers were not provided.^33–35^ Where reported, medians, IQR and variance were converted to mean and SD as suggested previously.^36^ It was not possible to convert all data to include in the meta-analysis; reasons for this were presenting an activity score/index with insufficient information to convert it into min/day,^37–46^ presenting an infeasible number of hours of activity (mean of 25 hours/day),^47^ summing multiple measurements annually^48^ and presenting energy expenditure per unit of body weight.^49^ A further seven studies did not report SD at one or more waves so the studies could not be included in the meta-analysis.^21 50–55^

### Statistical analysis

Data for each adolescent-adulthood comparison were combined in STATA using a random effects meta-analysis (STATA V.14). As all studies included in the meta-analysis were converted to a common metric, non-standardised weighted mean differences (WMDs) were calculated. Variation attributable to heterogeneity was assessed using the I^2^ statistic. If heterogeneity (I^2^ >50%) was seen,^18^ meta-regression was used to test the impact of potential effect modifiers (baseline age, time between baseline and follow-up (the mean calculated for the adolescent and adult time periods used), region of study, baseline date, accelerometer vs non-accelerometer data, extent of conversion necessary to estimate MVPA). Effect modifiers were selected based on the data extracted and those used in a previous review.^18^ Risk of bias score was not investigated as a potential effect modifier due to limited variability. Age and time between measurements were calculated using mean ages reported (and/or time between measurements where relevant). Region of study was summarised into four categories: Europe, North America, lower-income and middle-income countries (LMICs; eg, Brazil and Iran) and other high income countries (eg, Australia and Japan). Baseline measurement date was taken from the included papers and referred to that used as baseline in this review where possible (other waves of data may have been available outside our inclusion criteria) but was not reported for nine studies.^33 34 38 40 47 49 56 57^ Variables that were associated (p<0.05) in single models were included in a multiple model to explore the variance that could be explained.

Due to sex-differences in biology and behaviour during adolescence,^58^ a post hoc decision was made to stratify meta-analyses by sex where data were available. In addition, due to the high heterogeneity of the data, a further post hoc meta-analysis was conducted only including accelerometer-assessed physical activity. For completeness, a separate meta-analysis including non-accelerometer methods was also conducted.

Lastly, the main meta-analysis was repeated restricting the age of baseline to ≤15 years old and ≥18 years old at follow-up to provide an estimate of change between more discrete age periods.

Funnel plot asymmetry and Eggers test for bias were conducted for all meta-analyses to investigate various forms of potential publication bias.

The results are reported in accordance with relevant guidance (Meta-Analysis of Observational Studies in Epidemiology (MOOSE)^59^ and Preferred reporting items for systematic reviews and meta-analyses: the PRISMA statement (PRISMA)^60^).

## Results

**Figure 1:**
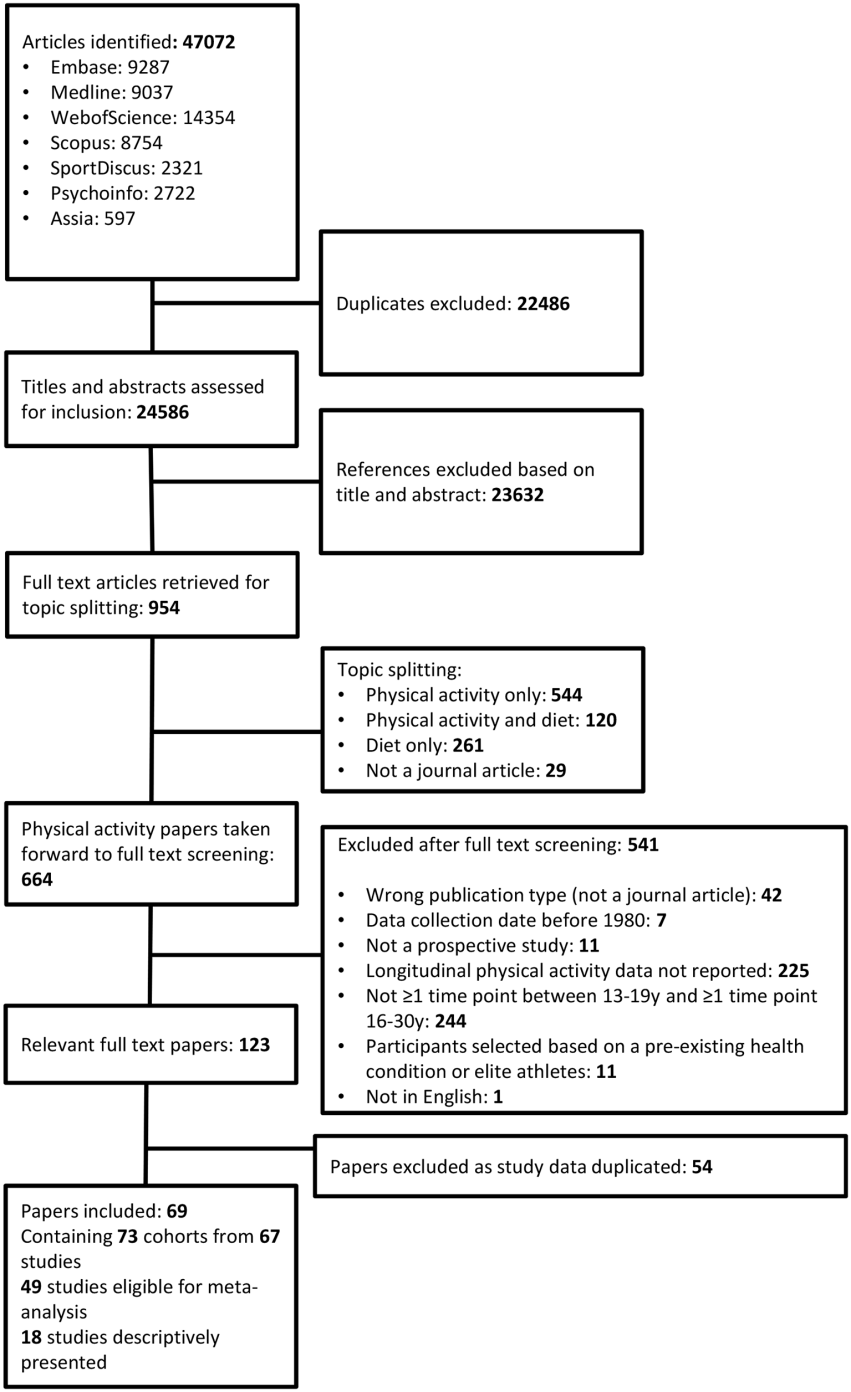
Evidence search and exclusion process.

Of 47 072 articles identified (including physical activity and diet papers), 22 486 duplicates were excluded. The remaining 24 586 had titles and abstracts assessed for inclusion; 23 632 papers were excluded based on title/abstract. Of the remaining 954 papers, 544 included physical activity only and 120 included both physical activity and diet and were taken forward to full-text screening. Of those full texts screened, 541 were excluded with the reasons included in figure 1; this left 123 full-text papers. Of these, 52 full-text papers were excluded as they included duplicate data available in other included papers. This left 69 papers containing data on 73 cohorts (defined as a specific group followed over time; some papers include multiple cohorts) from 67 overarching studies; data from 49 of these studies were eligible for meta-analysis. Study characteristics for all included studies are available in online supplementary table 3. Descriptive characteristics of the included studies, presented as those that were meta-analysed and those that were descriptively summarised are shown in table 2.

**Table 2:** Descriptive characteristics of the included studies, presented as those that were meta-analysed and those that were descriptively summarised (n=67 studies)

	Meta-analysed	Narrative
	N reporting (/49)	N reporting (/18)
Sample size (n)		
<100	9	7
100–499	22	6
500–999	7	1
>1000	11	4
Age at baseline (n) Mean (SD) years	15.6 (1.4)	15.8 (1.5)
Length of follow-up (n) Mean (SD) years	3.4 (2.6)	3.8 (3.0)
Region (n)		
Europe	20	10
North America	22	5
LMIC	3	0
Other HIC	4	3
Ethnicity (n) (% white Mean(SD))	20 69.3 (26.6)	5 72.1 (16.3)
**SES (n)** (% mothers with college degree Mean(SD))	9 39.7 (16.4)	2 46.5 (12.0)

SES expressed as % mothers with college degree (the most commonly reported measure).

HIC, high-income countries; LMIC, low-income and middle-income countries; SES, socioeconomic status.

Online supplementary table 4 presents the results of individual item and overall risk of bias assessment scores. Initially, 87% agreement was achieved on risk of bias scoring with discrepancies resolved via discussion. Of the 71 papers included, 2 received a ‘Strong’ rating, 3 (4%) were rated as ‘Moderate’ and 66 (93%) as ‘Weak’. The large number of ‘Weak’ scores was mainly due to the strict criteria of any single item scoring as ‘Weak’ meaning that the overall rating for that paper was ‘Weak’.

### Meta-analyses

**Figure 2:**
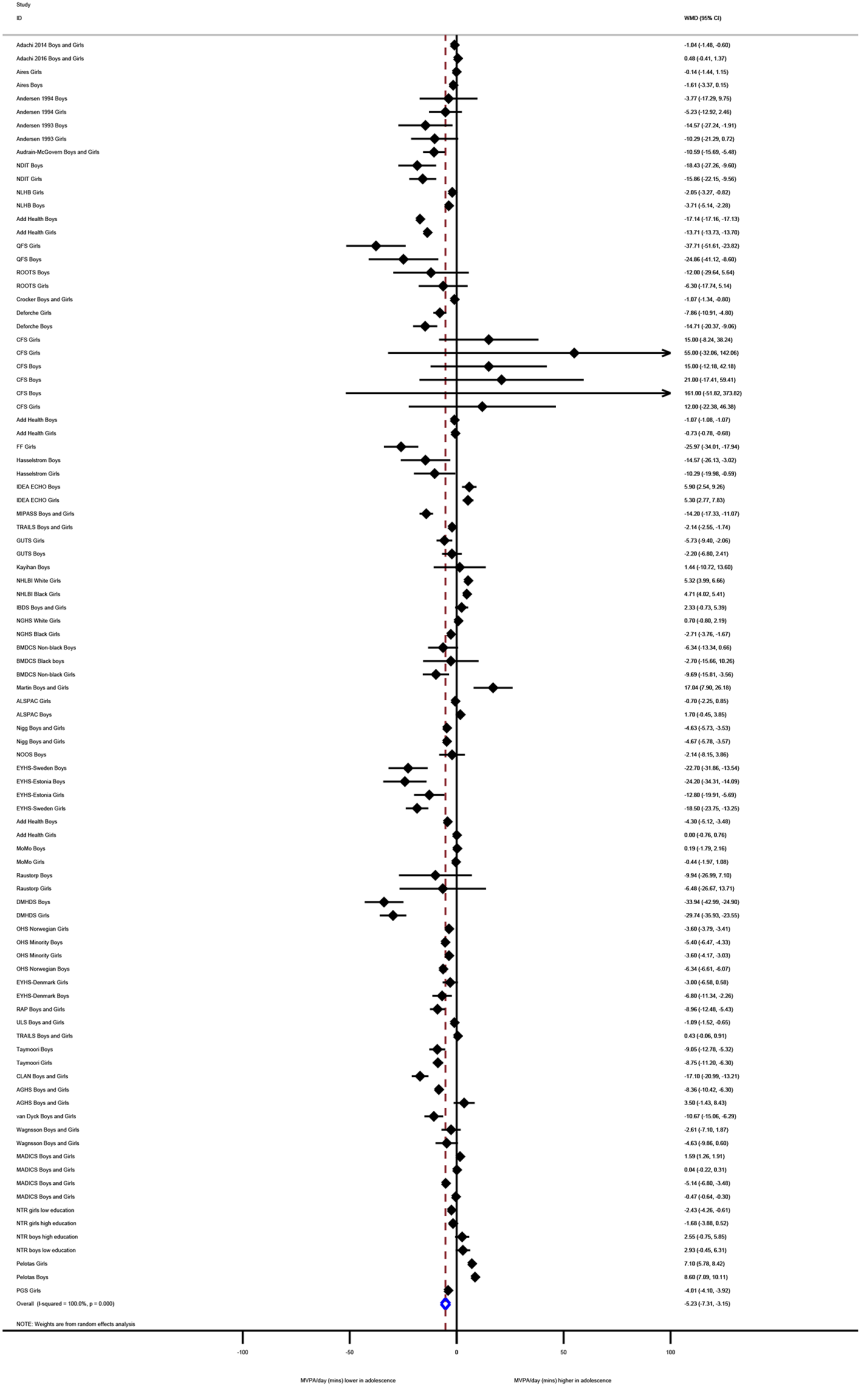
Change in physical activity (MVPA min/day) from adolescence to adulthood from all eligible studies. MVPA, moderate-to-vigorous physical activity.

The pooled WMDs displayed in table 3 and figure 2 indicate that physical activity is lower during adulthood than during adolescence WMD (95% CI) −5.2 (−7.3 to –3.1) min/day MVPA over a mean 3.4 (SD: 2.6) years. There was a statistically significant (χ2=5.8e+06, p<0.001) high amount of heterogeneity present between studies (I^2^ >99.0%), and none of the included predictors explained this variation (all p>0.05).

**Table 3:** Summary of physical activity reported in meta-analysed studies and meta-analysis results

Type of study included	N studies	Age range adolescence	Age range adulthood	Mean (SD) follow-up (years)	Mean (SD) adolescent MVPA min/day	Pooled WMD (95% CI)	I^2^ (%)
All eligible studies	49	13–19	16–30	3.4 (2.6)	41.0 (29.1)	−5.2 (−7.3 to -3.1)	>99.0
All eligible studies (males)	29	13–19	16–30	4.2 (2.8)	49.1 (31.1)	−6.5 (−10.6 to -2.3)	>99.0
All eligible studies (females)	30	13–19	16–30	3.8 (2.8)	37.2 (27.8)	−5.5 (−8.4 to -2.6)	>99.0
Accelerometer only	9	13–19	16–30	4.3 (2.9)	43.5 (16.8)	−7.4 (−11.6 to -3.1)	95.0
Non-accelerometer	40	13–19	16–30	3.2 (2.5)	40.4 (31.4)	−4.8 (−7.0 to -2.5)	>99.0
Restricted ages	3	13–15	18–30	7.4 (3.4)	47.9 (15.0)	−8.2 (−23.8 to 7.5)	89.7

Restricted ages: restricting the age of baseline to ≤15 years old and ≥18 years old at follow-up.

MVPA, moderate-to-vigorous physical activity; WMD, weighted mean difference.

Results for males and females (table 3 and see online supplementary figures 1 and 2, respectively) both indicated a decline in physical activity from adolescence to adulthood, both with very high heterogeneity between studies.

10.1136/bjsports-2016-097330.supp2 Supplementary file 2

**Figure 3:**
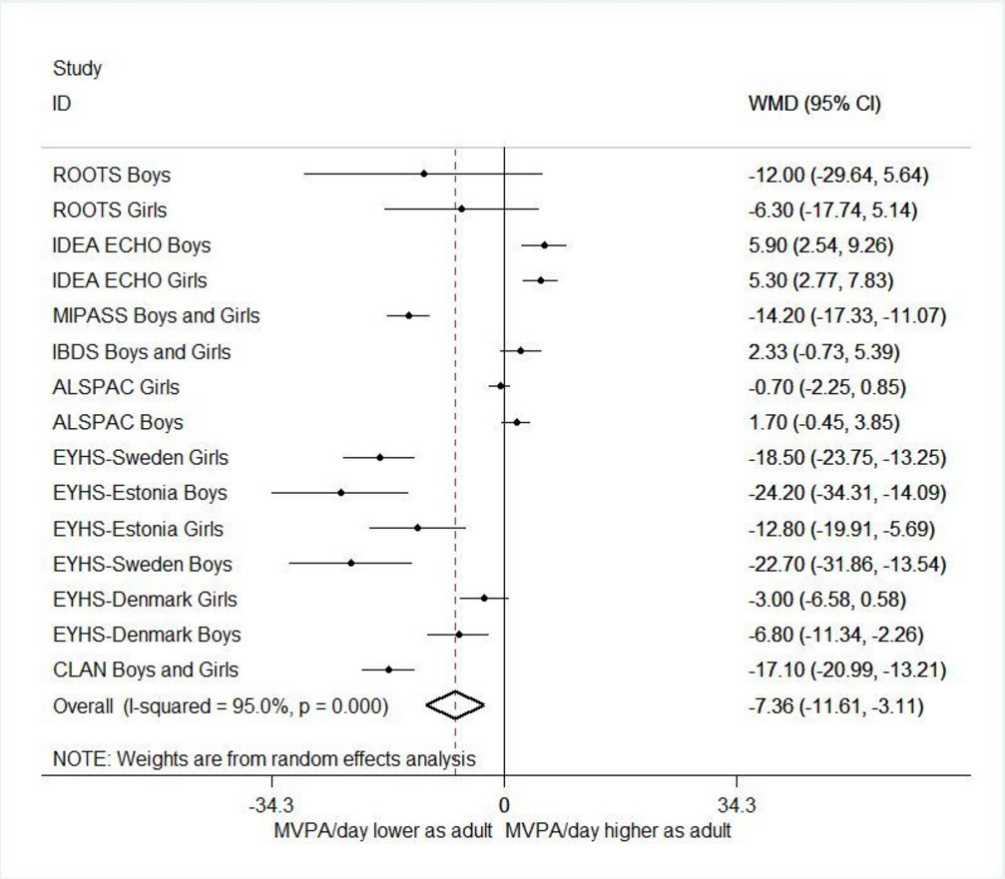
Change in physical activity (MVPA min/day) from adolescence to adulthood from eligible studies using accelerometer-assessed physical activity.

The results based only on accelerometer data suggested a slightly greater decline in MVPA between adolescence and adulthood −7.4 (−11.6 to –3.1) min/day MVPA with a high amount of heterogeneity present between studies (χ2=272.47, p<0.001, I^2^=95.0%) (table 3, figure 3). The only variable included in meta-regression that explained any of this variation was the difference in age between time-points with a greater age difference indicating more of a decline: WMD −1.9 (−3.6 to –0.2), p=0.03, with 27.0% of variation between studies explained. This value suggests that for every extra year of follow-up the decline is 1.9 min greater. When restricting to non-accelerometer data, the WMD was −6.3 (−8.1 to –4.4) min/day MVPA and none of the high amount of heterogeneity present between studies (χ2=5541.7, p<0.001, I^2^=99.2%) was explained by variables tested with meta-regression.

When adolescence was restricted to ≤15 years-old and adulthood as ≥18 years-old, three studies were included.^28 61 62^ When restricted to these distinct age groups, results were non-significant, but the extent of the WMD was comparable with the previous results (WMD −8.2 (−23.8 to 7.5) min/day MVPA); heterogeneity remained high (χ2=38.9, p<0.001, I^2^=89.7%) (table 3).

Funnel plot asymmetry and Eggers test for bias suggested no evidence of asymmetry in any meta-analyses (all p>0.188), indicating that these tests showed no evidence of publication bias.

Of the 18 studies that were not eligible for meta-analysis, nine statistically tested for change in physical activity over time; of these nine studies, seven showed a decline in physical activity^37 41 48 52^ and two showed no change between adolescence and adulthood.^21 47^ All of these studies used questionnaire reported measurement methods with the exception of one using an activity diary^49^ and one using a recall.^55^ The median (IQR) age in adolescence was 15.9 (14.9–16.5) years and 19.5 (17.4–22.0) years in adulthood. Median time between baseline and follow-up measurements was 2.0 (1.4–7.0) years and baseline participant numbers were 166 (84–994).

## Discussion

Meta-analysis of published longitudinal data indicated that physical activity declines over the transition from adolescence to adulthood. Results from all 49 eligible studies indicated that daily MVPA declined by a mean of 5.2 min/day, which is equivalent to approximately 13% of the mean baseline value; this decline occurred over a mean duration of 3.4 (SD: 2.6) years. Stratified analyses showed slight sex differences, with a slightly greater decline observed in males. For studies assessing physical activity with accelerometers, a decline of 7.4 min of daily MVPA was observed. This equated to 17% of the mean baseline estimate and occurred over a mean follow-up of 4.3 (SD: 2.9) years. Half of the non-meta-analysed studies statistically tested change in physical activity between adolescence and adulthood; of those, the majority (78%) reported a decline.

### Interpretation

All meta-analyses displayed large heterogeneity. This limits the validity of pooling the results and estimating a single summary result; the results of these meta-analyses should therefore be interpreted with caution. However, random effects meta-analysis should allow for heterogeneity and to some extent have led to relatively conservative results with wider CIs and larger p values when heterogeneity exists.

We investigated potential sources of heterogeneity using meta-regression and by separately reporting results for studies reporting accelerometer-assessed physical activity and self-report assessed activity. It was only in the accelerometer-assessed analysis that any variables investigated in meta-regression explained any variation, with a greater age difference between adolescence and adulthood indicating slightly more of a decline in physical activity as would be expected due to there being more time for change to occur.

The slightly larger decline observed in males than females could be at least partly because boys were more physically active than girls in adolescence. Previous work has observed a larger physical activity decline in girls during early adolescence (<12 years old) but a greater decrease among boys later in adolescence,^7 58^ suggesting a reduction in sex differences in physical activity later in adolescence. Our results add to this evidence by suggesting that these sex differences may further reduce over the transition to adulthood.

Although the inclusion criteria for early adulthood required at least one measurement of physical activity between the ages of 16 and 30, the majority of these data points were at the younger end of this age range. The adulthood measure in 35% of all studies was over age 21 with only 8% over 25 years old. For studies using accelerometers to measure physical activity, 33% (3/9) had a follow-up after age 21 with 11% (1/9) after 25 years old.

The tool we used to assess risk of bias led to only 2 of 71 (2.8%) papers being scored as strong; 66 (93%) were scored as weak. Our criteria were strict as just one ‘weak’ rating leading to the overall classification as ‘Weak’, Nevertheless, this suggests a paucity of good quality data that is important to further investigate due to the potential importance of early adulthood for developing long lasting health behaviours.

As adolescence and early adulthood are times of increasing autonomy, positive behaviours established during this time could potentially last into later adulthood, making this transitional time a potentially important target for health promotion.^63^ Furthermore, moving from adolescence into adulthood is characterised by transitions between different social roles^14^ and major transitions including moving out of the family home, changes in school/work environment and financial circumstances. Many of these transitions are associated with health behaviours including physical activity, but further work would be useful to characterise physical activity over the young adulthood period while taking account of these transitions.^11^ The period of late adolescence to early adulthood has been suggested as an important, but overlooked, age to establish long-term health behaviour patterns.^63^

### Relationship to prior knowledge

Although our results indicate that MVPA appears to decline by as much as 7 min/day between adolescence and early adulthood, the physical activity recommendations for adolescents (60 min/day MVPA) and adults (150 min/week of moderate or 75 min/week of vigorous activity) differ by more than this. As a weekly average, the adult recommendations suggest 21 min of moderate, or 10 min of vigorous activity daily, which is substantially less than the 60 min/day recommended in adolescence. Therefore, the absolute change in activity identified here of up to 7 min/day does not appear to account for the difference between adolescent (60 min/day) and adult physical activity recommendations (~10–21 min/day).

One could therefore argue that this decline is acceptable and should not present a health hazard at a population level. However, when this decline is put in the context of a meta-analysis from a large worldwide study^2^ indicating that a 10 min differencein MVPA was associated with a smaller waist circumference and lower fasting insulin among young people, this decline may have important health implications at the population level. Furthermore, the mean time between adolescence and adulthood measurements was 3.4 years, and although we did not test the linearity of changes over time, if this change was extrapolated over 10 years (eg, 15–25 years), it would amount to a potential decline of closer to 15 min/day. Further research with objective measures in high-quality studies that continue well into adulthood with multiple time-points are needed to further investigate this. Although we did not assess tracking of activity here, low physical activity in adolescence is likely to progress to adulthood inactivity,^35^ which is linked to increased risk of many diseases including type 2 diabetes, cancer and also mortality.^9^

The evidence on changes in physical activity during other stages of the life course suggests declines of 4% per year throughout childhood^64^ and 7% per year during adolescence.^7^ The modest decline observed here is comparable in relative terms, equating to an approximate 13% decline compared with the baseline value over a mean follow-up duration of 3.4 years. Taken together, this evidence suggests that at a population level, physical activity declines steadily throughout the first decades of life and that preventing declines in physical activity throughout this period could be valuable. Studying population-level estimates may mask individual variations in patterns of change and the important influence of various social transitions that may occur at different times for different people (such as starting employment, moving out of the family home or having children). Further research on trajectories of change in physical activity behaviour may help unravel some of these uncertainties.

### Strengths and limitations

To our knowledge, this is the first study to meta-analyse change in physical activity over the transition between adolescence and adulthood. Although heterogeneity was high and results should be interpreted with caution, limiting the results to studies using accelerometer-assessed physical activity, which should reduce measurement error,^65^ indicated a similar decline. Despite the high heterogeneity, we believe that the review would be less useful without the meta-analyses. Although we investigated potential causes of heterogeneity using meta-regression, few factors were significant.

In the analysis focused on accelerometer-assessed activity, there was some indication that time between measurements may have explained some of the heterogeneity. We acknowledge slight deviations from our published protocol. We did not search in the Cochrane library (we excluded trials), CINAHL (not relevant as is nursing journal database) and Psychlit (not available at our institution). However, as reference checking of included papers did not identify additional papers, we are confident that we have done a comprehensive search. We did not conduct duplicate abstract screening, which is a limitation, although 1500 articles were triple screened to ensure consistency among the three reviewers. Furthermore, although data extraction was 100% checked that it was not extracted in duplicate.

We believe that defining adolescence as 13–19 years old is appropriate. Although menarche may occur for some girls before age 13, socially, especially in developed countries (source of 95% of the data) 13 years old is classed as at least mid-adolescence. Our analysis design distinguishes this review as we focused on the transition out of adolescence into adulthood rather than change in physical activity during adolescence, which has been reviewed previously.^7 16^ As we used overlapping age groups, an included study may have two time-points that are arguably both in adolescence (such as 13 and 17 years old). The sensitivity analysis restricting to studies reporting data on <15 years old (adolescence) and >18 years old (adulthood) indicated a non-significant decline of −8.2 (−23.8, 7.5) min/day MVPA, but with only three studies included with this strict age criteria, we believe using broader age categories was appropriate to be as inclusive as possible.

A strength of this work is that sufficient homogenous data were present to perform a meta-analysis. However, data conversion was necessary, requiring assumptions that may have led to error in the absolute values used, although this should not have greatly influenced the within-study change estimates. Similarly, we used the data presented in each paper that was most similar to MVPA. Sometimes this was not directly comparable (eg, overall activity) but was converted to min/day. As most studies did not statistically test change in physical activity and only provided descriptive data, this conversion and meta-analysis was useful to gain a tangible overview of results.

The review aimed to characterise change in physical activity between adolescence and adulthood, therefore we did not use all time-points separately as they were condensed into broad adolescent and adulthood age periods; annual change has been examined throughout adolescence^7^ and was beyond the scope of this review. We were keen to take study size into account so we believe it was most appropriate to use meta-analysis, rather than computing an estimate such as percentage annual change. The included studies were all published in English and mostly from North America, Europe and other high-income countries (95% of studies), so these results are not likely to be generalisable to LMICs. We only included peer-reviewed publications, which may be susceptible to publication bias; however, funnel plots did not indicate evidence of asymmetry.

### Implications for policy, practice and research

Physical activity declines between adolescence and adulthood. Average adult physical activity levels are too low to benefit a range of health outcomes. Continued efforts to maintain or increase adolescent physical activity and to prevent a decline into adulthood could have important public health benefits. More high-quality longitudinal physical activity data over this transition would be valuable, especially in the young adulthood period, to better characterise physical activity patterns over this time to inform how physical activity promotion interventions might most effectively target this important group.

## Conclusion

Published longitudinal data indicate that physical activity declines over the transition from adolescence to adulthood by an average of 5.2 min of MVPA per day. Both males and females had lower physical activity in adulthood compared with adolescence. Objectively assessed physical activity showed a slightly larger decline of 7.4 min of daily MVPA. More objective longitudinal physical activity data over this transition would be valuable, as would investigating how change in physical activity is associated with various contemporaneous social transitions.

**What is already known?**

- Physical activity is lower in adulthood than adolescence.
- The adolescence–adulthood transition is important to target behaviour change interventions due to increasing autonomy, and positive behaviours established over this time have the potential to last into later adulthood.

**What are the findings?**

- Data from 49 studies indicate that over the transition from adolescence to adulthood moderate-to-vigorous physical activity (MVPA) declines by a mean of 5.2 min/day (or approximately 13% of the baseline value).
- Most studies assessed physical activity by self-report, but the nine studies that used accelerometers showed a mean decline of 7.4 min of daily MVPA (17% of mean baseline value) between adolescence and adulthood.
- The observed decline in physical activity was slightly larger in males than females (−6.5 vs −5.5 min/day) and, based on the accelerometer data, the decline was larger with each year of follow-up (further decline of −1.9 min/day per year).

10.1136/bjsports-2016-097330.supp3 Supplementary file 3
