# Supplementary material for: Change in physical activity from adolescence to early adulthood: a systematic review and meta-analysis of longitudinal cohort studies
Source: Br J Sports Med. 2017 Jul 24;53(8):496–503. doi: 10.1136/bjsports-2016-097330 (PMC6250429; doi:10.1136/bjsports-2016-097330)
Supplement: Supplementary file 1 [file bjsports-2016-097330supp001.docx]

Supplementary Table 1: Search terms

| 1 | Diet Outcomes  Physical activity outcomes | OR “Exercise”[MeSH] OR “Sports”[Mesh] OR "physical activity"[Title/Abstract] OR "physical activities"[Title/Abstract] OR “physically active”[Title/Abstract] OR "active transport"[Title/Abstract] OR "active travel"[Title/Abstract] OR exercise*[Title/Abstract] OR cycle[Title/Abstract] OR cycling[Title/Abstract] OR walk*[Title/Abstract] OR sport*[Title/Abstract] OR "energy expenditure"[Title/Abstract]  OR food*[Title/Abstract] OR nutrient*[Title/Abstract] OR macronutrient*[Title/Abstract] OR “energy intake”[Title/Abstract] OR diet[Title/Abstract] OR diets[Title/Abstract] OR “dietary”[Title/Abstract] OR nutrition[Title/Abstract] OR nutritional[Title/Abstract] OR fruit[Title/Abstract] OR vegetable[Title/Abstract] OR fruits[Title/Abstract] OR vegetables[Title/Abstract] OR snack*[Title/Abstract] OR “soft drink*”[Title/Abstract] OR soda[Title/Abstract] OR SSB[Title/Abstract] OR SSBs[Title/Abstract] OR salt[Title/Abstract] OR sugar*[Title/Abstract] OR "Food"[Mesh] OR "Beverages"[Mesh] OR diet[Mesh] OR "Nutrition Surveys"[Mesh] OR "Diet Records"[Mesh] OR "Dietary Fats"[Mesh] OR "Dietary Proteins"[Mesh] OR "Dietary Carbohydrates "[Mesh] OR "Micronutrients"[Mesh] |
| --- | --- | --- |
| 2 | Longitudinal | longitudinal[Title/Abstract] OR cohort[Title/Abstract] OR prospective[Title/Abstract] OR “follow-up stud*”[Title/Abstract] OR “follow up stud*”[Title/Abstract] OR tracking[Title/Abstract] OR "Follow-Up Studies"[Mesh] OR “Prospective Studies”[Mesh] OR “Longitudinal Studies”[Mesh] |
| 3 | Age range | adolescent*[Title/Abstract] OR adolescence[Title/Abstract] OR teen*[Title/Abstract] OR student*[Title/Abstract] OR “young adult*”[Title/Abstract] OR “young adulthood”[Title/Abstract] OR “early adulthood”[Title/Abstract] OR “emerging adulthood”[Title/Abstract] OR youth*[Title/Abstract] OR “young people”[Title/Abstract] OR freshman[Title/Abstract] OR freshmen[Title/Abstract] |
| 4 | Additional filters | English[lang]  Restrict to publication year 1980 or after |
| 5 |  | 1 AND 2 AND 3 AND 4 |

The search strategy was originally designed for PubMed and then adapted as necessary for the other databases.

Supplementary Table 2. Risk of bias scoring criteria, adapted from the Effective Public Health Practice Project (EPHPP) Quality Assessment Tool

| **Characteristic** | **Question** | **Scoring** | **Scoring** |
| --- | --- | --- | --- |
| **Representativeness** | Are the individuals selected to participate in the study likely to be representative of the target population? | 1 Very likely  2 Somewhat  3 Not likely  4 Can’t tell | Strong  Moderate  Weak  Weak |
|  | What percentage of selected individuals agreed to participate? | 1 80-100%  2 60–79%  3 <60%  4 Not applicable  5 Can’t tell | Strong  Moderate  Weak  Weak  Weak |
| **Number of participants** | How many participants were in the study? | 1 >1000  2 999-101  3 <100 | Strong  Moderate  Weak |
| **Drop-outs** | Were withdrawals and drop-outs reported in terms of numbers and/or reasons per group? | 1 Yes  2 No  3 Can’t tell  4 Not Applicable | Strong  Moderate  Weak  Weak |
|  | Indicate the percentage of participants completing the study. (If the percentage differs by groups, record the lowest). | 1 80-100%  2 60–79%  3 <60%  4 Not applicable  5 Can’t tell | Strong  Moderate  Weak  Weak  Weak |
| **Data collection** | Was the tool objective or subjective? | 1 Objective  2 Reported  3 Can’t tell | Strong  Moderate/Weak  Weak |
|  | Was the tool valid? | 1 Yes  2 No  3 Can’t tell | Strong/Moderate  Weak  Weak |
|  | Was the tool reliable? | 1 Yes  2 No  3 Can’t tell | Strong/Moderate  Weak  Weak |
|  | Was the tool the same at all time-points? | 1 Yes  2 No  3 Can’t tell | Strong-Weak  Weak |
| **Analyses** | Was change in physical activity statistically tested? | 1 Yes  2 No  3 Can’t tell | Strong/Moderate  Weak  Weak |
|  | Was adjustment for potential confounders included? | 1 Yes  2 No  3 Can’t tell | Strong/Moderate  Moderate/Weak  Weak |

When multiple questions represent one category, the results of all category questions were combined to obtain a score and the lowest ranking for a category was taken. For example, if a self-reported measure of activity was reported to be valid and reliable and the same over both time-points, it was scored as ‘Moderate’. If a self-reported measure of activity was not reported to be valid and reliable or was different over time-points it was scored as ‘Weak’.

Scores for each item were summed and the score was defined as ‘Weak’ when at least one item was classed as ‘Weak’. Papers were classed as ‘Strong’ when three out of the five criteria were rated as ‘Strong’ and no items were scored as ‘Weak’; other studies were classed as ‘Moderate’.

Supplementary Table 3. Descriptive characteristics of included papers

| **Paper** | **Study name** | **Country** | **Date** | **N** | **% boys** | **Ethnicity** | **SES** | **Baseline mean age (y)** | **Follow-up mean ages (y)** | **Assessment** | **Meta-analysed** |
| --- | --- | --- | --- | --- | --- | --- | --- | --- | --- | --- | --- |
| Adachi et al., 2014 [[66](#_ENREF_66)] | - | Canada | - | 1771 | 49.2 | 92% Canadian | High | 13 (10mo) | 15.5*, 16.5*, 17.5* | Questionnaire | Yes |
|  |  |  |  |  |  |  |  |  |  |  |  |
| Adachi et al., 2016 [[33](#_ENREF_33)] | - | Canada | - | 1132 | 29.4 | 88% Canadian | High | 19.0 (0.9) | 20 | Questionnaire | Yes |
|  |  |  |  |  |  |  |  |  |  |  |  |
| Aires et al., 2012 [[67](#_ENREF_67)] | - | Portugal | 05 | 170 | 42.9 | - | - | 15.2 (1.3) | 16.2 (1.3) | Questionnaire index | Yes |
|  |  |  |  |  |  |  |  |  |  |  |  |
| Andersen et al., 1993 [[68](#_ENREF_68)] | - | Denmark | 83 | 305 | 43.6 | - | - | 17 | 25 | Questionnaire | Yes |
|  |  |  |  |  |  |  |  |  |  |  |  |
| Andersen et al., 1994 [[69](#_ENREF_69)] | - | Denmark | 83 | 307 | 38.1 | - | - | 16.5 (0.6) | 18.5 | Questionnaire | Yes |
|  |  |  |  |  |  |  |  |  |  |  |  |
| Audrain-McGovern et al., 2012 [[57](#_ENREF_57)] | - | USA | - | 1384 | 50 | 73% white | 25-65% parents ≥college | 14 | 16, 17.5 | Questionnaire | Yes |
|  |  |  |  |  |  |  |  |  |  |  |  |
| Bagur-Calafat et al., 2015 [[47](#_ENREF_47)] | - | Spain | - | 6 | 0 | - | - | 14 | 15*, 16* | Questionnaire | No |
|  |  |  |  |  |  |  |  |  |  |  |  |
| Barnett et al., 2013 [[70](#_ENREF_70)] | NDIT | Canada | 01 | 951 | 48.6 | - | 52% ≥1 parent ≥degree | Boys 15.2 (0.4)  Girls 15.1 (0.4) | Boys 17.0 (0.4)  Girls 16.9 (0.4) | Questionnaire | Yes |
|  |  |  |  |  |  |  |  |  |  |  |  |
| Baxter-Jones et al., 2008 [[48](#_ENREF_48)] | PBMAS | Canada | 91 | 369 | 100+ | 98% white | - | 15.3 (1.0) | 16.1 (1.0)  17.1 (0.9) | Questionnaire index | No |
|  |  |  |  |  |  |  |  |  |  |  |  |
| Benitez-Porres et al., 2016 [[44](#_ENREF_44)] | - | Spain | 11 | 80 | 47.5 | - | - | Boys 14.6 (2.6)  Girls 14.5 (1.8) | Boys 15.1 (2.4); 16.1 (2.3)  Girls 14.8 (1.7); 16.2 (1.6) | Questionnaire index | No |
|  |  |  |  |  |  |  |  |  |  |  |  |
| Birkeland et al., 2009 [[71](#_ENREF_71)] | NLHB | Norway | 92 | 945 | 55 | - | 20% low, 54% middle, 26% high | 15 | 16, 18, 19, 21, 23 | Questionnaire | Yes |
|  |  |  |  |  |  |  |  |  |  |  |  |
| Boone-Heinonen et al., 2010 [[72](#_ENREF_72)] | Add Health | USA | 94 | 12701 | 49.1 | >68% white | 14.7% boys, 15.2% girls’ parents <high school | Boys: 15.5 (0.1)  Girls: 15.3 (0.1) | Boys: 21.9 (0.1)  Girls 21.7 (0.1) | Interview~ | Yes |
|  |  |  |  |  |  |  |  |  |  |  |  |
| Boreham et al., 2004 [[37](#_ENREF_37)] | YH | UK | 92 | 476 | 51.5 | - | - | 15 | 22.0 (0.6) | Questionnaire index | No |
|  |  |  |  |  |  |  |  |  |  |  |  |
| Campbell et al., 2001 [[73](#_ENREF_73)] | QFS | Canada | 80 | 145 | 49.7 | - | - | Boys 13.5 (2.4)  Girls 13.4 (2.6) | Boys 25.3 (3.5)  Girls 25.4 (3.4) | 3 day diary | Yes |
|  |  |  |  |  |  |  |  |  |  |  |  |
| Collings et al., 2015 [[74](#_ENREF_74)] | ROOTS | UK | 06 | 144 | 50 | - | - | 15.1 (0.3) | Boys 17.4 (0.3)  Girls 17.5 (0.3) | Actiheart accelerometer | Yes |
|  |  |  |  |  |  |  |  |  |  |  |  |
| Crocker et al., 2003 [[75](#_ENREF_75)] | - | Canada | 98 | 705 | 0 | - | Mixed | 15.5* | 16.5* | Questionnaire | Yes |
|  |  |  |  |  |  |  |  |  |  |  |  |
| de Souza et al., 2015 [[38](#_ENREF_38)] | OGHPS | Portugal | - | 959 | 47.2 | - | - | 17 | 18 | Questionnaire index | No |
|  |  |  |  |  |  |  |  |  |  |  |  |
| Deforche et al., 2015 [[76](#_ENREF_76)] | - | Belgium | 08 | 2726 | 33.3 | - | 15.7% mothers ≥degree | 17.3 (0.5) | 18.8 | Questionnaire | Yes |
|  |  |  |  |  |  |  |  |  |  |  |  |
| Deheeger et al., 2002 [[50](#_ENREF_50)] | LSNG | France | 97 | 92 | 59.8 | - | - | 14 | 16 | Questionnaire | No |
|  |  |  |  |  |  |  |  |  |  |  |  |
| Eime et al., 2016 [[55](#_ENREF_55)] | - | Australia | 08 | 84 | 0 | - | - | 16.2 (0.6) | 17.2 | Recall | No |
| Fortier et al., 2001 [[62](#_ENREF_62)] | CFS | Canada | 81 | 88 | 60 | - | - | 13.5* | 20.5* | Questionnaire | Yes |
|  |  |  |  |  |  |  |  | 15.5* | 22.5* |  |  |
|  |  |  |  |  |  |  |  | 17.5* | 25.5* |  |  |
|  |  |  |  |  |  |  |  |  |  |  |  |
| Freitas et al., 2012 [[39](#_ENREF_39)] | MGS | Portugal | 96 | 170 | 50 | - | - | 15.9 (0.3) | 23 | Questionnaire | No |
|  |  |  |  |  |  |  |  |  |  |  |  |
| Gordon-Larsen et al., 2001 [[77](#_ENREF_77)] | Add Health | USA | 95 | 12759 | 49.3 | 56.4% white | - | 15.9 (0.1) | 16.9 | Questionnaire | Yes |
|  |  |  |  |  |  |  |  |  |  |  |  |
| Graham et al., 2011 [[54](#_ENREF_54)] | EAT | USA | 98 | 1902 | 0 | 53.5% white | - | 14.9 (1.6) | 19.9, 24.9 | Questionnaire | No |
|  |  |  |  |  |  |  |  |  |  |  |  |
| Gunnell et al., (2016) [[45](#_ENREF_45)] | REAL | Canada | 06 | 1072 | 42.7 | 74.1% white | 54.9% both parents ≥college | 13.5 (1.1) | 14.7 (1.36); 16.11 (1.45); 17.2 (1.4) | Questionnaire | No |
| Han et al., 2008 [[78](#_ENREF_78)] | FF | USA | 04 | 171 | 0 | 84% white | - | 18.2 (0.4) | 19.2 | Questionnaire | Yes |
|  |  |  |  |  |  |  |  |  |  |  |  |
| Hasselstrom et al., 2002 [[79](#_ENREF_79)] | - | Denmark | 80 | 305 | 43.6 | - | - | 17.1 (1.0) | 25.1 | Questionnaire | Yes |
|  |  |  |  |  |  |  |  |  |  |  |  |
| Hearst et al., 2012 [[80](#_ENREF_80)] | IDEA ECHO | USA | 06 | 578 | 49.7 | 86.9% white | 78% parents ≥college | 14.6 (1.8) | 16.6 | Actigraph accelerometer | Yes |
|  |  |  |  |  |  |  |  |  |  |  |  |
| Hobin et al., 2014 [[81](#_ENREF_81)] | MIPASS | Canada | 08 | 447 | 45.6 | - | - | 15.2 (0.8) | 18.7* | Actical (and Actigraph^#^) accelerometer | Yes |
|  |  |  |  |  |  |  |  |  |  |  |  |
| Hunter et al., 2016 [[82](#_ENREF_82)] | COMPASS | Canada | 13 | 18777 | 46.4 | 73.7% white | 35% students have>$20 spending money/week | 15.1 (0.02) | 16.1 | Questionnaire | Yes |
| Huppertz et al., 2016 [[46](#_ENREF_46)] | FinnTwin12 | Finland | 97 | 3977 | 49.9 | - | 38% mothers with high education | 14.04 (0.08) to 14.05 (0.09) | 16.89 to 17.62 | Questionnaire | No |
| Huppertz et al., 2016 [[46](#_ENREF_46)] | NTR | Netherlands | 00 | 8162 | 44.0 | Mainly white | 34% mothers with university education | 14.63 (0.6) | 16.9 | Questionnaire | Yes |
| Janssens et al., 2014 [[83](#_ENREF_83)] | TRAILS | Netherlands | 89 | 1661 | 47.9 | - | - | 16.3 (0.7) | 19.1 (0.6) | Questionnaire | Yes |
|  |  |  |  |  |  |  |  |  |  |  |  |
| Jung et al., 2008 [[21](#_ENREF_21)] | - | Canada | 02 | 133 | 0 | 68% white, 11% Asian | - | 18.5 (0.6) | 19.5* | Questionnaire | No |
|  |  |  |  |  |  |  |  |  |  |  |  |
| Kahn et al., 2008 [[84](#_ENREF_84)] | GUTS | USA | 97 | 215 | 33.5 | Largely white | - | 15 | 16, 17 | Questionnaire | Yes |
|  |  |  |  |  |  |  |  |  |  |  |  |
| Kayihan et al., 2014 [[56](#_ENREF_56)] | - | Turkey | - | 94 | 100 | - | - | 18 | 22 | Questionnaire | Yes |
|  |  |  |  |  |  |  |  |  |  |  |  |
| Kimm et al., 2002 [[22](#_ENREF_22)] | NGHS | USA | 87 | 2379 | 0 | 51% black, 49% white | 21.1% black, 49.6% white parents ≥college | 15.5* | 16.5*, 17.5, 18.5, 23.5 | Questionnaire | Yes |
|  |  |  |  |  |  |  |  |  |  |  |  |
| Kwon et al., 2015 [[85](#_ENREF_85)] | NGHS | USA | 87 | 2155 | 0 | 49% white | 19% white, 30% black parents <high school | 14 | 16.5 | Questionnaire | Yes |
|  |  |  |  |  |  |  |  |  |  |  |  |
| Kwon et al., 2015 [[28](#_ENREF_28)] | IBDS | USA | 98 | 467 | 49.9 | - | 72.2% mothers ≥college | 13 | 15, 17, 19 | Actigraph accelerometer | Yes |
|  |  |  |  |  |  |  |  |  |  |  |  |
| Lantz et al., 2008 [[49](#_ENREF_49)] | - | Sweden | - | 186 | 46.3 | - | - | 15 | 20.5 | 7 day diary | No |
|  |  |  |  |  |  |  |  |  |  |  |  |
| Lappe et al., 2014 [[23](#_ENREF_23)] | BMDCS | USA | 02 |  | 52 | 23% black | - | 14.1 | 17.5 | Questionnaire | Yes |
|  |  |  |  |  |  |  |  | 13.1 | 14.4, 17.5 |  |  |
|  |  |  |  |  |  |  |  | 13.1 | 16.4 |  |  |
|  |  |  |  |  |  |  |  |  |  |  |  |
| Lemoyne et al., 2016 [[86](#_ENREF_86)] | - | Canada | 08 | 195 | 35 | - | - | 16.3 | 17.8 (2.4) | Questionnaire | Yes |
| Magarey et al., 1999 [[40](#_ENREF_40)] | Adelaide | Australia | - | 106 | 50.9 | - | - | 15 | 17 | Questionnaire index | No |
|  |  |  |  |  |  |  |  |  |  |  |  |
| Martin et al., 2010 [[87](#_ENREF_87)] | - | Australia | 06 | 213 | 38 | 93% speak English | 84% in education | 17.0 (0.9) | 18.0 (0.9) | Questionnaire | Yes |
|  |  |  |  |  |  |  |  |  |  |  |  |
|  |  |  |  |  |  |  |  |  |  |  |  |
| Mitchell et al., 2012 [[88](#_ENREF_88)] | ALSPAC | UK | 19 | 1341 | 44.7 | - | 8% mothers no qualifications | 14 | 16 | Actigraph accelerometer | Yes |
|  |  |  |  |  |  |  |  |  |  |  |  |
| Nigg 2001 [[89](#_ENREF_89)] | - | USA | 95 | 819 | 54.8 | - | - | 14.9 (1.2) | 17.6 (1.2) | Questionnaire index | Yes |
|  |  |  |  |  |  |  |  |  |  |  |  |
| Nordstrom et al., 2008 [[34](#_ENREF_34)] | NOOS | Sweden | - | 27 | 100 | - | - | 17.1 (1.7) | 24.8 (1.9) | Questionnaire | Yes |
|  |  |  |  |  |  |  |  |  |  |  |  |
| Ortega et al., 2013 [[61](#_ENREF_61)] | EYHS Sweden | Sweden | 98 | 360 | 43.6 | 88% white | 35.7% boys, 21.1% girls’ mothers ≥university | 15.6 (0.4) | Boys 21 (0.4)  Girls 21 (0.7) | Actigraph accelerometer | Yes |
|  |  |  |  |  |  |  |  |  |  |  |  |
| Ortega et al., 2013 [[61](#_ENREF_61)] | EYHS Estonia | Estonia | 98 | 379 | 41.2 | 100% white | 29.6% boys, 30.4% girls’ mothers ≥university | Boys 15.5 (0.6)  Girls 15.3 (0.5) | Boys 25.3 (0.5)  Girls 25.1 (0.5) | Actigraph accelerometer | Yes |
|  |  |  |  |  |  |  |  |  |  |  |  |
| Palakshappa et al., 2015 [[25](#_ENREF_25)] | Add Health | USA | 96 | 1774 | 49.3 | 60.9% white | 5.8% <high school | 16* | 28* | Questionnaire index~ | Yes |
|  |  |  |  |  |  |  |  |  |  |  |  |
| Pinto et al., 1998 [[51](#_ENREF_51)] | - | USA | 98 | 332 | 40 | 67% white | - | 18.6 (1.8) | 19.6* | Questionnaire | No |
|  |  |  |  |  |  |  |  |  |  |  |  |
| Porkka et al., 1997 [[41](#_ENREF_41)] | CRYF | Finland | 80 | 1054 | - | - | - | 16.5* | 19.5, 22.5, 25.5*, 28.5* | Questionnaire index | No |
|  |  |  |  |  |  |  |  |  |  |  |  |
| Ramires et al., 2016 [[90](#_ENREF_90)] | Pelotas | Brazil | 08 | 4324 | 48.8 | 12.6% white | 20% in richest quintile | 15 | 18 | Questionnaire | Yes |
| Rauner et al., 2015 [[42](#_ENREF_42)] | MoMo | Germany | 09 | 818 | 47.2 | - | - | 15.5* | 21.5* | Questionnaire | No |
| Raustorp et al., 2013 [[35](#_ENREF_35)] | - | Sweden | 03 | 40 | 52.5 | - | Middle class | Boys 15.5 (0.8)  Girls 15.9 (0.8) | Boys 17.5 (0.8) 22.5 (0.8)  Girls 17.9 (0.8) 22.9 (0.8) | Pedometer | Yes |
|  |  |  |  |  |  |  |  |  |  |  |  |
| Richards et al., 2009 [[91](#_ENREF_91)] | DMHDS | New Zealand | 87 | 832 | 51 | - | Mixed | 15 | 18 | Questionnaire | Yes |
|  |  |  |  |  |  |  |  |  |  |  |  |
| Rockette-Wagner et al., 2016 [[92](#_ENREF_92)] | PGS | USA | 10 | 832 | 0 | 38.4% white | 34.8% receiving public assistance | 15.5 | 16.5 | Pedometer | Yes |
| Sagatun et al., 2008 [[26](#_ENREF_26)] | OHS | Norway | 00 | 3811 | 29.2 | 20% minority | - | 15.5* | 18 | Questionnaire | Yes |
|  |  |  |  |  |  |  |  |  |  |  |  |
| Schipperijn et al., 2015 [[93](#_ENREF_93)] | EYHS Denmark | Denmark | 03 | 177 | 42.9 | - | - | Boys 15.7 (0.3)  Girls 15.7 (0.4) | Boys 21.8 (0.3)  Girls 21.7 (0.4) | Actigraph accelerometer | Yes |
|  |  |  |  |  |  |  |  |  |  |  |  |
| Shi et al., 2006 [[52](#_ENREF_52)] | - | Japan | 98 | 96 | 47.9 | - | - | 16.5 (0.3) | 17.5 (0.3) 18.3 (0.3) | Questionnaire | No |
|  |  |  |  |  |  |  |  |  |  | Questionnaire | |
| Simons et al., 2015 [[94](#_ENREF_94)] | RAP | Australia | 03 | 440 | 50.9 | - | - | 17.6 (0.6) | 18.6, 19.6 | Questionnaire | Yes |
|  |  |  |  |  |  |  |  |  |  |  |  |
| Small et al., 2012 [[95](#_ENREF_95)] | ULS | USA | 07 | 716 | 49.2 | 25% Hispanic | - | 18.4 (0.4) | 19.4 | Questionnaire | Yes |
|  |  |  |  |  |  |  |  |  |  |  |  |
| Stavrakakis et al., 2012 [[96](#_ENREF_96)] | TRAILS | Netherlands | 03 | 2149 | 49 | - | - | 13.7 (0.5) | 16.3 (0.7) | Questionnaire | Yes |
|  |  |  |  |  |  |  |  |  |  |  |  |
| Taymoori et al., 2011 [[97](#_ENREF_97)] | - | Iran | 06 | 1073 | 48 | - | - | 14.4 (1.6) | 16.4 (1.7) | 6 day diary | Yes |
|  |  |  |  |  |  |  |  |  |  |  |  |
| Telama et al., 2014 [[43](#_ENREF_43)] | YFS | Finland | 92 | 374 | 48 | - | - | 18 | 27 | Questionnaire | No |
|  |  |  |  | 419 | 53.2 | - | - | 15 | 24, 30 |  |  |
|  |  |  |  |  |  |  |  |  |  |  |  |
| Telford et al., 2012 [[98](#_ENREF_98)] | CLAN | Australia | 04 | 259 | - | - | - | 14.5(0.6) | 16.3 (0.6) | Actigraph accelerometer | Yes |
|  |  |  |  |  |  |  |  |  |  |  |  |
| Van de Laar et al., 2010 [[99](#_ENREF_99)] | AGHS | Netherlands | 80 | 373 | 47.5 | - | - | 16 | 21, 27 | Interview | Yes |
|  |  |  |  |  |  |  |  |  |  |  |  |
| Van Dyck et al., 2014 [[100](#_ENREF_100)] | - | Belgium | 08 | 291 | 33.3 | - | 26.5% mothers <college | 17.2 (0.5) | 18.7 | Interview | Yes |
|  |  |  |  |  |  |  |  |  |  |  |  |
| Wagnsson et al., 2014 [[101](#_ENREF_101)] | - | Sweden | 05 | 439 | 59 | 90% Swedish | - | 14* | 15* | Questionnaire | Yes |
|  | | | | 454 | 59 | 90% Swedish | - | 16.5* |  |  |  |
|  |  |  |  |  |  |  |  |  |  |  |  |
| Wichstrom et al., 2013 [[53](#_ENREF_53)] | YIN | Norway | 92 | 3251 | - | - | - | 16* | 17.5* | Questionnaire | No |
|  |  |  |  |  |  |  |  |  |  |  |  |
| Zarrett et al., 2014 [[27](#_ENREF_27)] | MADICS | USA | 93 | 1037 | 49 | 66% African 30% white | - | 12.8 (2.0) | 16.5 | Questionnaire | Yes |

Date of baseline data collection: last two digits of year e.g. 00 represents 2000

*estimated from other data in the paper

^#^ only at baseline

~different physical activity measure at baseline and follow-up

AT: active transport

SP: leisure time sports participation

+only boys eligible based on age groupings

NR: not reported

Supplementary Table 4: Risk of bias assessment scores

| **Paper** | **Rate selection** | **Rate N** | **Rate drop out** | **Rate tool** | **Rate analyses** | **Overall rating** |
| --- | --- | --- | --- | --- | --- | --- |
| Adachi et al., 2016 [[33](#_ENREF_33)] | Moderate | Strong | Moderate | Weak | Weak | Weak |
| Adachi et al., 2014 [[66](#_ENREF_66)] | Moderate | Strong | Moderate | Weak | Weak | Weak |
| Aires et al., 2012 [[67](#_ENREF_67)] | Weak | Moderate | Weak | Weak | Weak | Weak |
| Andersen et al., 1994 [[69](#_ENREF_69)] | Moderate | Moderate | Strong | Weak | Moderate | Weak |
| Andersen et al., 1993 [[68](#_ENREF_68)] | Moderate | Moderate | Strong | Weak | Moderate | Weak |
| Audrain-McGovern et al., 2012 [[57](#_ENREF_57)] | Moderate | Strong | Strong | Moderate | Moderate | Moderate |
| Bagur-Calafat et al., 2015 [[47](#_ENREF_47)] | Weak | Weak | Strong | Weak | Moderate | Weak |
| Barnett et al., 2013 [[70](#_ENREF_70)] | Weak | Strong | Strong | Weak | Strong | Weak |
| Baxter-Jones et al., 2008 [[48](#_ENREF_48)] | Weak | Moderate | Weak | Moderate | Weak | Weak |
| Benitez-Porres et al., 2016 [[44](#_ENREF_44)] | Weak | Moderate | Moderate | Moderate | Weak | Weak |
| Bielemann et al., 2014 #6519} | Moderate | Strong | Weak | Weak | Weak | Weak |
| Birkeland et al., 2009 [[71](#_ENREF_71)] | Moderate | Moderate | Moderate | Weak | Weak | Weak |
| Boone-Heinonen et al., 2010 [[72](#_ENREF_72)] | Moderate | Strong | Moderate | Weak | Weak | Weak |
| Boreham et al., 2004 [[37](#_ENREF_37)] | Weak | Moderate | Weak | Weak | Weak | Weak |
| Campbell et al., 2001 [[73](#_ENREF_73)] | Weak | Weak | Weak | Moderate | Weak | Weak |
| Collings et al., 2015 [[74](#_ENREF_74)] | Weak | Moderate | Weak | Strong | Strong | Weak |
| Crocker et al., 2003 [[75](#_ENREF_75)] | Weak | Moderate | Strong | Moderate | Moderate | Weak |
| de Souza et al., 2015 [[38](#_ENREF_38)] | Weak | Moderate | Weak | Moderate | Weak | Weak |
| Deforche et al., 2015 [[76](#_ENREF_76)] | Weak | Moderate | Weak | Moderate | Moderate | Weak |
| Deheeger et al., 2002 [[50](#_ENREF_50)] | Weak | Weak | Weak | Weak | Weak | Weak |
| Eime et al., 2016 [[55](#_ENREF_55)] | Weak | Moderate | Moderate | Weak | Weak | Weak |
| Fortier et al., 2001 [[62](#_ENREF_62)] | Weak | Weak | Weak | Moderate | Weak | Weak |
| Freitas et al., 2012 [[39](#_ENREF_39)] | Weak | Moderate | Weak | Moderate | Weak | Weak |
| Gordon-Larsen et al., 2001 [[77](#_ENREF_77)] | Moderate | Strong | Moderate | Weak | Weak | Weak |
| Graham et al., 2011 [[54](#_ENREF_54)] | Weak | Strong | Weak | Weak | Weak | Weak |
| Gunnell et al., (2016) [[45](#_ENREF_45)] | Weak | Weak | Moderate | Strong | Weak | Weak |
| Han et al., 2008 [[78](#_ENREF_78)] | Weak | Weak | Moderate | Moderate | Moderate | Weak |
| Hasselstrom et al., 2002 [[79](#_ENREF_79)] | Strong | Moderate | Moderate | Weak | Weak | Weak |
| Hearst et al., 2012 [[80](#_ENREF_80)] | Weak | Strong | Strong | Strong | Strong | Strong |
| Hobin et al., 2014 [[81](#_ENREF_81)] | Weak | Moderate | Moderate | Moderate | Moderate | Weak |
| Hunter et al., 2016 [[82](#_ENREF_82)] | Moderate | Weak | Moderate | Weak | Weak | Moderate |
| Huppertz et al., 2016 [[46](#_ENREF_46)] | Weak | Weak | Weak | Weak | Weak | Weak |
| Janssens et al., 2014 [[83](#_ENREF_83)] | Moderate | Strong | Moderate | Weak | Weak | Weak |
| Jung et al., 2008 [[21](#_ENREF_21)] | Weak | Moderate | Moderate | Weak | Moderate | Weak |
| Kahn et al., 2008 [[84](#_ENREF_84)] | Moderate | Moderate | Weak | Moderate | Strong | Weak |
| Kayihan et al., 2014 [[56](#_ENREF_56)] | Weak | Weak | Weak | Moderate | Moderate | Weak |
| Kimm et al., 2002 [[22](#_ENREF_22)] | Moderate | Strong | Strong | Weak | Strong | Weak |
| Kwon et al., 2015 [[85](#_ENREF_85)] | Weak | Moderate | Weak | Strong | Strong | Weak |
| Kwon et al., 2015 [[28](#_ENREF_28)] | Moderate | Strong | Strong | Moderate | Weak | Weak |
| Lantz et al., 2008 [[49](#_ENREF_49)] | Weak | Weak | Weak | Weak | Weak | Weak |
| Lappe et al., 2014 [[23](#_ENREF_23)] | Weak | Strong | Weak | Weak | Weak | Weak |
| Lemoyne et al., 2016 [[86](#_ENREF_86)] | Weak | Weak | Weak | Weak | Weak | Weak |
| Magarey et al., 1999 [[40](#_ENREF_40)] | Weak | Moderate | Weak | Weak | Weak | Weak |
| Martin et al., 2010 [[87](#_ENREF_87)] | Weak | Moderate | Weak | Moderate | Weak | Weak |
| Martinez-Gomez et al., 2014 [[24](#_ENREF_24)] | Strong | Strong | Moderate | Weak | Weak | Weak |
| Mitchell et al., 2012 [[88](#_ENREF_88)] | Weak | Strong | Weak | Strong | Weak | Weak |
| Nigg 2001 [[89](#_ENREF_89)] | Weak | Moderate | Weak | Moderate | Moderate | Weak |
| Nordstrom et al., 2008 [[34](#_ENREF_34)] | Weak | Weak | Weak | Weak | Weak | Weak |
| Ortega et al., 2013 [[61](#_ENREF_61)] | Weak | Moderate | Weak | Strong | Strong | Weak |
| Palakshappa et al., 2015 [[25](#_ENREF_25)] | Weak | Strong | Weak | Weak | Strong | Weak |
| Pinto et al., 1998 [[51](#_ENREF_51)] | Weak | Moderate | Moderate | Weak | Weak | Weak |
| Porkka et al., 1997 [[41](#_ENREF_41)] | Moderate | Moderate | Weak | Weak | Moderate | Weak |
| Ramires et al., 2016 [[90](#_ENREF_90)] | Weak | Strong | Weak | Weak | Weak | Weak |
| Rauner et al., 2015 [[42](#_ENREF_42)] | Moderate | Moderate | Weak | Moderate | Moderate | Weak |
| Raustorp et al., 2013 [[35](#_ENREF_35)] | Weak | Weak | Weak | Strong | Moderate | Weak |
| Richards et al., 2009 [[91](#_ENREF_91)] | Weak | Moderate | Strong | Moderate | Strong | Weak |
| Rockette-Wagner et al., 2016 [[92](#_ENREF_92)] | Strong | Strong | Strong | Strong | Strong | Strong |
| Sagatun et al., 2008 [[26](#_ENREF_26)] | Moderate | Strong | Moderate | Moderate | Strong | Moderate |
| Schipperijn et al., 2015 [[93](#_ENREF_93)] | Weak | Moderate | Weak | Strong | Weak | Weak |
| Shi et al., 2006 [[52](#_ENREF_52)] | Weak | Weak | Moderate | Weak | Weak | Weak |
| Simons et al., 2015 [[94](#_ENREF_94)] | Weak | Moderate | Moderate | Weak | Strong | Weak |
| Small et al., 2012 [[95](#_ENREF_95)] | Moderate | Strong | Strong | Weak | Strong | Weak |
| Stavrakakis et al., 2012 [[96](#_ENREF_96)] | Weak | Strong | Strong | Weak | Weak | Weak |
| Taymoori et al., 2011 [[97](#_ENREF_97)] | Weak | Strong | Moderate | Moderate | Moderate | Weak |
| Telama et al., 2014 [[43](#_ENREF_43)] | Strong | Moderate | Weak | Weak | Weak | Weak |
| Telford et al., 2012 [[98](#_ENREF_98)] | Weak | Moderate | Weak | Strong | Weak | Weak |
| van de Laar et al., 2010 [[99](#_ENREF_99)] | Weak | Moderate | Weak | Weak | Weak | Weak |
| van Dyck et al., 2014 [[100](#_ENREF_100)] | Moderate | Moderate | Weak | Weak | Strong | Weak |
| Wagnsson et al., 2014 [[101](#_ENREF_101)] | Moderate | Moderate | Moderate | Weak | Moderate | Weak |
| Wichstrom et al., 2013 [[53](#_ENREF_53)] | Weak | Strong | Weak | Weak | Weak | Weak |
| Zarrett et al., 2014 [[27](#_ENREF_27)] | Moderate | Strong | Moderate | Weak | Weak | Weak |
