# Supplementary material for: Change in physical activity from adolescence to early adulthood: a systematic review and meta-analysis of longitudinal cohort studies
Source: Br J Sports Med. 2017 Jul 24;53(8):496–503. doi: 10.1136/bjsports-2016-097330 (PMC6250429; doi:10.1136/bjsports-2016-097330)
Supplement: Supplementary file 3 [file bjsports-2016-097330supp003.docx]

Supplementary Table 1: Search terms

| 1 | Diet Outcomes  Physical activity outcomes | OR “Exercise”[MeSH] OR “Sports”[Mesh] OR "physical activity"[Title/Abstract] OR "physical activities"[Title/Abstract] OR “physically active”[Title/Abstract] OR "active transport"[Title/Abstract] OR "active travel"[Title/Abstract] OR exercise*[Title/Abstract] OR cycle[Title/Abstract] OR cycling[Title/Abstract] OR walk*[Title/Abstract] OR sport*[Title/Abstract] OR "energy expenditure"[Title/Abstract]  OR food*[Title/Abstract] OR nutrient*[Title/Abstract] OR macronutrient*[Title/Abstract] OR “energy intake”[Title/Abstract] OR diet[Title/Abstract] OR diets[Title/Abstract] OR “dietary”[Title/Abstract] OR nutrition[Title/Abstract] OR nutritional[Title/Abstract] OR fruit[Title/Abstract] OR vegetable[Title/Abstract] OR fruits[Title/Abstract] OR vegetables[Title/Abstract] OR snack*[Title/Abstract] OR “soft drink*”[Title/Abstract] OR soda[Title/Abstract] OR SSB[Title/Abstract] OR SSBs[Title/Abstract] OR salt[Title/Abstract] OR sugar*[Title/Abstract] OR "Food"[Mesh] OR "Beverages"[Mesh] OR diet[Mesh] OR "Nutrition Surveys"[Mesh] OR "Diet Records"[Mesh] OR "Dietary Fats"[Mesh] OR "Dietary Proteins"[Mesh] OR "Dietary Carbohydrates "[Mesh] OR "Micronutrients"[Mesh] |
| --- | --- | --- |
| 2 | Longitudinal | longitudinal[Title/Abstract] OR cohort[Title/Abstract] OR prospective[Title/Abstract] OR “follow-up stud*”[Title/Abstract] OR “follow up stud*”[Title/Abstract] OR tracking[Title/Abstract] OR "Follow-Up Studies"[Mesh] OR “Prospective Studies”[Mesh] OR “Longitudinal Studies”[Mesh] |
| 3 | Age range | adolescent*[Title/Abstract] OR adolescence[Title/Abstract] OR teen*[Title/Abstract] OR student*[Title/Abstract] OR “young adult*”[Title/Abstract] OR “young adulthood”[Title/Abstract] OR “early adulthood”[Title/Abstract] OR “emerging adulthood”[Title/Abstract] OR youth*[Title/Abstract] OR “young people”[Title/Abstract] OR freshman[Title/Abstract] OR freshmen[Title/Abstract] |
| 4 | Additional filters | English[lang]  Restrict to publication year 1980 or after |
| 5 |  | 1 AND 2 AND 3 AND 4 |

The search strategy was originally designed for PubMed and then adapted as necessary for the other databases.

Supplementary Table 2. Risk of bias scoring criteria, adapted from the Effective Public Health Practice Project (EPHPP) Quality Assessment Tool

| **Characteristic** | **Question** | **Scoring** | **Scoring** |
| --- | --- | --- | --- |
| **Representativeness** | Are the individuals selected to participate in the study likely to be representative of the target population? | 1 Very likely  2 Somewhat  3 Not likely  4 Can’t tell | Strong  Moderate  Weak  Weak |
|  | What percentage of selected individuals agreed to participate? | 1 80-100%  2 60–79%  3 <60%  4 Not applicable  5 Can’t tell | Strong  Moderate  Weak  Weak  Weak |
| **Number of participants** | How many participants were in the study? | 1 >1000  2 999-101  3 <100 | Strong  Moderate  Weak |
| **Drop-outs** | Were withdrawals and drop-outs reported in terms of numbers and/or reasons per group? | 1 Yes  2 No  3 Can’t tell  4 Not Applicable | Strong  Moderate  Weak  Weak |
|  | Indicate the percentage of participants completing the study. (If the percentage differs by groups, record the lowest). | 1 80-100%  2 60–79%  3 <60%  4 Not applicable  5 Can’t tell | Strong  Moderate  Weak  Weak  Weak |
| **Data collection** | Was the tool objective or subjective? | 1 Objective  2 Reported  3 Can’t tell | Strong  Moderate/Weak  Weak |
|  | Was the tool valid? | 1 Yes  2 No  3 Can’t tell | Strong/Moderate  Weak  Weak |
|  | Was the tool reliable? | 1 Yes  2 No  3 Can’t tell | Strong/Moderate  Weak  Weak |
|  | Was the tool the same at all time-points? | 1 Yes  2 No  3 Can’t tell | Strong-Weak  Weak |
| **Analyses** | Was change in physical activity statistically tested? | 1 Yes  2 No  3 Can’t tell | Strong/Moderate  Weak  Weak |
|  | Was adjustment for potential confounders included? | 1 Yes  2 No  3 Can’t tell | Strong/Moderate  Moderate/Weak  Weak |

When multiple questions represent one category, the results of all category questions were combined to obtain a score and the lowest ranking for a category was taken. For example, if a self-reported measure of activity was reported to be valid and reliable and the same over both time-points, it was scored as ‘Moderate’. If a self-reported measure of activity was not reported to be valid and reliable or was different over time-points it was scored as ‘Weak’.

Scores for each item were summed and the score was defined as ‘Weak’ when at least one item was classed as ‘Weak’. Papers were classed as ‘Strong’ when three out of the five criteria were rated as ‘Strong’ and no items were scored as ‘Weak’; other studies were classed as ‘Moderate’.

Supplementary Table 3. Descriptive characteristics of included papers

| **Paper** | **Study name** | **Country** | **Date** | **N** | **% boys** | **Ethnicity** | **SES** | **Baseline mean age (y)** | **Follow-up mean ages (y)** | **Assessment** | **Meta-analysed** |
| --- | --- | --- | --- | --- | --- | --- | --- | --- | --- | --- | --- |
| Adachi et al., 2014 [[1](#_ENREF_1)] | - | Canada | - | 1771 | 49.2 | 92% Canadian | High | 13 (10mo) | 15.5*, 16.5*, 17.5* | Questionnaire | Yes |
|  |  |  |  |  |  |  |  |  |  |  |  |
| Adachi et al., 2016 [[2](#_ENREF_2)] | - | Canada | - | 1132 | 29.4 | 88% Canadian | High | 19.0 (0.9) | 20 | Questionnaire | Yes |
|  |  |  |  |  |  |  |  |  |  |  |  |
| Aires et al., 2012 [[3](#_ENREF_3)] | - | Portugal | 05 | 170 | 42.9 | - | - | 15.2 (1.3) | 16.2 (1.3) | Questionnaire index | Yes |
|  |  |  |  |  |  |  |  |  |  |  |  |
| Andersen et al., 1993 [[4](#_ENREF_4)] | - | Denmark | 83 | 305 | 43.6 | - | - | 17 | 25 | Questionnaire | Yes |
|  |  |  |  |  |  |  |  |  |  |  |  |
| Andersen et al., 1994 [[5](#_ENREF_5)] | - | Denmark | 83 | 307 | 38.1 | - | - | 16.5 (0.6) | 18.5 | Questionnaire | Yes |
|  |  |  |  |  |  |  |  |  |  |  |  |
| Audrain-McGovern et al., 2012 [[6](#_ENREF_6)] | - | USA | - | 1384 | 50 | 73% white | 25-65% parents ≥college | 14 | 16, 17.5 | Questionnaire | Yes |
|  |  |  |  |  |  |  |  |  |  |  |  |
| Bagur-Calafat et al., 2015 [[7](#_ENREF_7)] | - | Spain | - | 6 | 0 | - | - | 14 | 15*, 16* | Questionnaire | No |
|  |  |  |  |  |  |  |  |  |  |  |  |
| Barnett et al., 2013 [[8](#_ENREF_8)] | NDIT | Canada | 01 | 951 | 48.6 | - | 52% ≥1 parent ≥degree | Boys 15.2 (0.4)  Girls 15.1 (0.4) | Boys 17.0 (0.4)  Girls 16.9 (0.4) | Questionnaire | Yes |
|  |  |  |  |  |  |  |  |  |  |  |  |
| Baxter-Jones et al., 2008 [[9](#_ENREF_9)] | PBMAS | Canada | 91 | 369 | 100+ | 98% white | - | 15.3 (1.0) | 16.1 (1.0)  17.1 (0.9) | Questionnaire index | No |
|  |  |  |  |  |  |  |  |  |  |  |  |
| Benitez-Porres et al., 2016 [[10](#_ENREF_10)] | - | Spain | 11 | 80 | 47.5 | - | - | Boys 14.6 (2.6)  Girls 14.5 (1.8) | Boys 15.1 (2.4); 16.1 (2.3)  Girls 14.8 (1.7); 16.2 (1.6) | Questionnaire index | No |
|  |  |  |  |  |  |  |  |  |  |  |  |
| Birkeland et al., 2009 [[11](#_ENREF_11)] | NLHB | Norway | 92 | 945 | 55 | - | 20% low, 54% middle, 26% high | 15 | 16, 18, 19, 21, 23 | Questionnaire | Yes |
|  |  |  |  |  |  |  |  |  |  |  |  |
| Boone-Heinonen et al., 2010 [[12](#_ENREF_12)] | Add Health | USA | 94 | 12701 | 49.1 | >68% white | 14.7% boys, 15.2% girls’ parents <high school | Boys: 15.5 (0.1)  Girls: 15.3 (0.1) | Boys: 21.9 (0.1)  Girls 21.7 (0.1) | Interview~ | Yes |
|  |  |  |  |  |  |  |  |  |  |  |  |
| Boreham et al., 2004 [[13](#_ENREF_13)] | YH | UK | 92 | 476 | 51.5 | - | - | 15 | 22.0 (0.6) | Questionnaire index | No |
|  |  |  |  |  |  |  |  |  |  |  |  |
| Campbell et al., 2001 [[14](#_ENREF_14)] | QFS | Canada | 80 | 145 | 49.7 | - | - | Boys 13.5 (2.4)  Girls 13.4 (2.6) | Boys 25.3 (3.5)  Girls 25.4 (3.4) | 3 day diary | Yes |
|  |  |  |  |  |  |  |  |  |  |  |  |
| Collings et al., 2015 [[15](#_ENREF_15)] | ROOTS | UK | 06 | 144 | 50 | - | - | 15.1 (0.3) | Boys 17.4 (0.3)  Girls 17.5 (0.3) | Actiheart accelerometer | Yes |
|  |  |  |  |  |  |  |  |  |  |  |  |
| Crocker et al., 2003 [[16](#_ENREF_16)] | - | Canada | 98 | 705 | 0 | - | Mixed | 15.5* | 16.5* | Questionnaire | Yes |
|  |  |  |  |  |  |  |  |  |  |  |  |
| de Souza et al., 2015 [[17](#_ENREF_17)] | OGHPS | Portugal | - | 959 | 47.2 | - | - | 17 | 18 | Questionnaire index | No |
|  |  |  |  |  |  |  |  |  |  |  |  |
| Deforche et al., 2015 [[18](#_ENREF_18)] | - | Belgium | 08 | 2726 | 33.3 | - | 15.7% mothers ≥degree | 17.3 (0.5) | 18.8 | Questionnaire | Yes |
|  |  |  |  |  |  |  |  |  |  |  |  |
| Deheeger et al., 2002 [[19](#_ENREF_19)] | LSNG | France | 97 | 92 | 59.8 | - | - | 14 | 16 | Questionnaire | No |
|  |  |  |  |  |  |  |  |  |  |  |  |
| Eime et al., 2016 [[20](#_ENREF_20)] | - | Australia | 08 | 84 | 0 | - | - | 16.2 (0.6) | 17.2 | Recall | No |
| Fortier et al., 2001 [[21](#_ENREF_21)] | CFS | Canada | 81 | 88 | 60 | - | - | 13.5* | 20.5* | Questionnaire | Yes |
|  |  |  |  |  |  |  |  | 15.5* | 22.5* |  |  |
|  |  |  |  |  |  |  |  | 17.5* | 25.5* |  |  |
|  |  |  |  |  |  |  |  |  |  |  |  |
| Freitas et al., 2012 [[22](#_ENREF_22)] | MGS | Portugal | 96 | 170 | 50 | - | - | 15.9 (0.3) | 23 | Questionnaire | No |
|  |  |  |  |  |  |  |  |  |  |  |  |
| Gordon-Larsen et al., 2001 [[23](#_ENREF_23)] | Add Health | USA | 95 | 12759 | 49.3 | 56.4% white | - | 15.9 (0.1) | 16.9 | Questionnaire | Yes |
|  |  |  |  |  |  |  |  |  |  |  |  |
| Graham et al., 2011 [[24](#_ENREF_24)] | EAT | USA | 98 | 1902 | 0 | 53.5% white | - | 14.9 (1.6) | 19.9, 24.9 | Questionnaire | No |
|  |  |  |  |  |  |  |  |  |  |  |  |
| Gunnell et al., (2016) [[25](#_ENREF_25)] | REAL | Canada | 06 | 1072 | 42.7 | 74.1% white | 54.9% both parents ≥college | 13.5 (1.1) | 14.7 (1.36); 16.11 (1.45); 17.2 (1.4) | Questionnaire | No |
| Han et al., 2008 [[26](#_ENREF_26)] | FF | USA | 04 | 171 | 0 | 84% white | - | 18.2 (0.4) | 19.2 | Questionnaire | Yes |
|  |  |  |  |  |  |  |  |  |  |  |  |
| Hasselstrom et al., 2002 [[27](#_ENREF_27)] | - | Denmark | 80 | 305 | 43.6 | - | - | 17.1 (1.0) | 25.1 | Questionnaire | Yes |
|  |  |  |  |  |  |  |  |  |  |  |  |
| Hearst et al., 2012 [[28](#_ENREF_28)] | IDEA ECHO | USA | 06 | 578 | 49.7 | 86.9% white | 78% parents ≥college | 14.6 (1.8) | 16.6 | Actigraph accelerometer | Yes |
|  |  |  |  |  |  |  |  |  |  |  |  |
| Hobin et al., 2014 [[29](#_ENREF_29)] | MIPASS | Canada | 08 | 447 | 45.6 | - | - | 15.2 (0.8) | 18.7* | Actical (and Actigraph^#^) accelerometer | Yes |
|  |  |  |  |  |  |  |  |  |  |  |  |
| Hunter et al., 2016 [[30](#_ENREF_30)] | COMPASS | Canada | 13 | 18777 | 46.4 | 73.7% white | 35% students have>$20 spending money/week | 15.1 (0.02) | 16.1 | Questionnaire | Yes |
| Huppertz et al., 2016 [[31](#_ENREF_31)] | FinnTwin12 | Finland | 97 | 3977 | 49.9 | - | 38% mothers with high education | 14.04 (0.08) to 14.05 (0.09) | 16.89 to 17.62 | Questionnaire | No |
| Huppertz et al., 2016 [[31](#_ENREF_31)] | NTR | Netherlands | 00 | 8162 | 44.0 | Mainly white | 34% mothers with university education | 14.63 (0.6) | 16.9 | Questionnaire | Yes |
| Janssens et al., 2014 [[32](#_ENREF_32)] | TRAILS | Netherlands | 89 | 1661 | 47.9 | - | - | 16.3 (0.7) | 19.1 (0.6) | Questionnaire | Yes |
|  |  |  |  |  |  |  |  |  |  |  |  |
| Jung et al., 2008 [[33](#_ENREF_33)] | - | Canada | 02 | 133 | 0 | 68% white, 11% Asian | - | 18.5 (0.6) | 19.5* | Questionnaire | No |
|  |  |  |  |  |  |  |  |  |  |  |  |
| Kahn et al., 2008 [[34](#_ENREF_34)] | GUTS | USA | 97 | 215 | 33.5 | Largely white | - | 15 | 16, 17 | Questionnaire | Yes |
|  |  |  |  |  |  |  |  |  |  |  |  |
| Kayihan et al., 2014 [[35](#_ENREF_35)] | - | Turkey | - | 94 | 100 | - | - | 18 | 22 | Questionnaire | Yes |
|  |  |  |  |  |  |  |  |  |  |  |  |
| Kimm et al., 2002 [[36](#_ENREF_36)] | NGHS | USA | 87 | 2379 | 0 | 51% black, 49% white | 21.1% black, 49.6% white parents ≥college | 15.5* | 16.5*, 17.5, 18.5, 23.5 | Questionnaire | Yes |
|  |  |  |  |  |  |  |  |  |  |  |  |
| Kwon et al., 2015 [[37](#_ENREF_37)] | NGHS | USA | 87 | 2155 | 0 | 49% white | 19% white, 30% black parents <high school | 14 | 16.5 | Questionnaire | Yes |
|  |  |  |  |  |  |  |  |  |  |  |  |
| Kwon et al., 2015 [[38](#_ENREF_38)] | IBDS | USA | 98 | 467 | 49.9 | - | 72.2% mothers ≥college | 13 | 15, 17, 19 | Actigraph accelerometer | Yes |
|  |  |  |  |  |  |  |  |  |  |  |  |
| Lantz et al., 2008 [[39](#_ENREF_39)] | - | Sweden | - | 186 | 46.3 | - | - | 15 | 20.5 | 7 day diary | No |
|  |  |  |  |  |  |  |  |  |  |  |  |
| Lappe et al., 2014 [[40](#_ENREF_40)] | BMDCS | USA | 02 |  | 52 | 23% black | - | 14.1 | 17.5 | Questionnaire | Yes |
|  |  |  |  |  |  |  |  | 13.1 | 14.4, 17.5 |  |  |
|  |  |  |  |  |  |  |  | 13.1 | 16.4 |  |  |
|  |  |  |  |  |  |  |  |  |  |  |  |
| Lemoyne et al., 2016 [[41](#_ENREF_41)] | - | Canada | 08 | 195 | 35 | - | - | 16.3 | 17.8 (2.4) | Questionnaire | Yes |
| Magarey et al., 1999 [[42](#_ENREF_42)] | Adelaide | Australia | - | 106 | 50.9 | - | - | 15 | 17 | Questionnaire index | No |
|  |  |  |  |  |  |  |  |  |  |  |  |
| Martin et al., 2010 [[43](#_ENREF_43)] | - | Australia | 06 | 213 | 38 | 93% speak English | 84% in education | 17.0 (0.9) | 18.0 (0.9) | Questionnaire | Yes |
|  |  |  |  |  |  |  |  |  |  |  |  |
|  |  |  |  |  |  |  |  |  |  |  |  |
| Mitchell et al., 2012 [[44](#_ENREF_44)] | ALSPAC | UK | 19 | 1341 | 44.7 | - | 8% mothers no qualifications | 14 | 16 | Actigraph accelerometer | Yes |
|  |  |  |  |  |  |  |  |  |  |  |  |
| Nigg 2001 [[45](#_ENREF_45)] | - | USA | 95 | 819 | 54.8 | - | - | 14.9 (1.2) | 17.6 (1.2) | Questionnaire index | Yes |
|  |  |  |  |  |  |  |  |  |  |  |  |
| Nordstrom et al., 2008 [[46](#_ENREF_46)] | NOOS | Sweden | - | 27 | 100 | - | - | 17.1 (1.7) | 24.8 (1.9) | Questionnaire | Yes |
|  |  |  |  |  |  |  |  |  |  |  |  |
| Ortega et al., 2013 [[47](#_ENREF_47)] | EYHS Sweden | Sweden | 98 | 360 | 43.6 | 88% white | 35.7% boys, 21.1% girls’ mothers ≥university | 15.6 (0.4) | Boys 21 (0.4)  Girls 21 (0.7) | Actigraph accelerometer | Yes |
|  |  |  |  |  |  |  |  |  |  |  |  |
| Ortega et al., 2013 [[47](#_ENREF_47)] | EYHS Estonia | Estonia | 98 | 379 | 41.2 | 100% white | 29.6% boys, 30.4% girls’ mothers ≥university | Boys 15.5 (0.6)  Girls 15.3 (0.5) | Boys 25.3 (0.5)  Girls 25.1 (0.5) | Actigraph accelerometer | Yes |
|  |  |  |  |  |  |  |  |  |  |  |  |
| Palakshappa et al., 2015 [[48](#_ENREF_48)] | Add Health | USA | 96 | 1774 | 49.3 | 60.9% white | 5.8% <high school | 16* | 28* | Questionnaire index~ | Yes |
|  |  |  |  |  |  |  |  |  |  |  |  |
| Pinto et al., 1998 [[49](#_ENREF_49)] | - | USA | 98 | 332 | 40 | 67% white | - | 18.6 (1.8) | 19.6* | Questionnaire | No |
|  |  |  |  |  |  |  |  |  |  |  |  |
| Porkka et al., 1997 [[50](#_ENREF_50)] | CRYF | Finland | 80 | 1054 | - | - | - | 16.5* | 19.5, 22.5, 25.5*, 28.5* | Questionnaire index | No |
|  |  |  |  |  |  |  |  |  |  |  |  |
| Ramires et al., 2016 [[51](#_ENREF_51)] | Pelotas | Brazil | 08 | 4324 | 48.8 | 12.6% white | 20% in richest quintile | 15 | 18 | Questionnaire | Yes |
| Rauner et al., 2015 [[52](#_ENREF_52)] | MoMo | Germany | 09 | 818 | 47.2 | - | - | 15.5* | 21.5* | Questionnaire | No |
| Raustorp et al., 2013 [[53](#_ENREF_53)] | - | Sweden | 03 | 40 | 52.5 | - | Middle class | Boys 15.5 (0.8)  Girls 15.9 (0.8) | Boys 17.5 (0.8) 22.5 (0.8)  Girls 17.9 (0.8) 22.9 (0.8) | Pedometer | Yes |
|  |  |  |  |  |  |  |  |  |  |  |  |
| Richards et al., 2009 [[54](#_ENREF_54)] | DMHDS | New Zealand | 87 | 832 | 51 | - | Mixed | 15 | 18 | Questionnaire | Yes |
|  |  |  |  |  |  |  |  |  |  |  |  |
| Rockette-Wagner et al., 2016 [[55](#_ENREF_55)] | PGS | USA | 10 | 832 | 0 | 38.4% white | 34.8% receiving public assistance | 15.5 | 16.5 | Pedometer | Yes |
| Sagatun et al., 2008 [[56](#_ENREF_56)] | OHS | Norway | 00 | 3811 | 29.2 | 20% minority | - | 15.5* | 18 | Questionnaire | Yes |
|  |  |  |  |  |  |  |  |  |  |  |  |
| Schipperijn et al., 2015 [[57](#_ENREF_57)] | EYHS Denmark | Denmark | 03 | 177 | 42.9 | - | - | Boys 15.7 (0.3)  Girls 15.7 (0.4) | Boys 21.8 (0.3)  Girls 21.7 (0.4) | Actigraph accelerometer | Yes |
|  |  |  |  |  |  |  |  |  |  |  |  |
| Shi et al., 2006 [[58](#_ENREF_58)] | - | Japan | 98 | 96 | 47.9 | - | - | 16.5 (0.3) | 17.5 (0.3) 18.3 (0.3) | Questionnaire | No |
|  |  |  |  |  |  |  |  |  |  | Questionnaire | |
| Simons et al., 2015 [[59](#_ENREF_59)] | RAP | Australia | 03 | 440 | 50.9 | - | - | 17.6 (0.6) | 18.6, 19.6 | Questionnaire | Yes |
|  |  |  |  |  |  |  |  |  |  |  |  |
| Small et al., 2012 [[60](#_ENREF_60)] | ULS | USA | 07 | 716 | 49.2 | 25% Hispanic | - | 18.4 (0.4) | 19.4 | Questionnaire | Yes |
|  |  |  |  |  |  |  |  |  |  |  |  |
| Stavrakakis et al., 2012 [[61](#_ENREF_61)] | TRAILS | Netherlands | 03 | 2149 | 49 | - | - | 13.7 (0.5) | 16.3 (0.7) | Questionnaire | Yes |
|  |  |  |  |  |  |  |  |  |  |  |  |
| Taymoori et al., 2011 [[62](#_ENREF_62)] | - | Iran | 06 | 1073 | 48 | - | - | 14.4 (1.6) | 16.4 (1.7) | 6 day diary | Yes |
|  |  |  |  |  |  |  |  |  |  |  |  |
| Telama et al., 2014 [[63](#_ENREF_63)] | YFS | Finland | 92 | 374 | 48 | - | - | 18 | 27 | Questionnaire | No |
|  |  |  |  | 419 | 53.2 | - | - | 15 | 24, 30 |  |  |
|  |  |  |  |  |  |  |  |  |  |  |  |
| Telford et al., 2012 [[64](#_ENREF_64)] | CLAN | Australia | 04 | 259 | - | - | - | 14.5(0.6) | 16.3 (0.6) | Actigraph accelerometer | Yes |
|  |  |  |  |  |  |  |  |  |  |  |  |
| Van de Laar et al., 2010 [[65](#_ENREF_65)] | AGHS | Netherlands | 80 | 373 | 47.5 | - | - | 16 | 21, 27 | Interview | Yes |
|  |  |  |  |  |  |  |  |  |  |  |  |
| Van Dyck et al., 2014 [[66](#_ENREF_66)] | - | Belgium | 08 | 291 | 33.3 | - | 26.5% mothers <college | 17.2 (0.5) | 18.7 | Interview | Yes |
|  |  |  |  |  |  |  |  |  |  |  |  |
| Wagnsson et al., 2014 [[67](#_ENREF_67)] | - | Sweden | 05 | 439 | 59 | 90% Swedish | - | 14* | 15* | Questionnaire | Yes |
|  | | | | 454 | 59 | 90% Swedish | - | 16.5* |  |  |  |
|  |  |  |  |  |  |  |  |  |  |  |  |
| Wichstrom et al., 2013 [[68](#_ENREF_68)] | YIN | Norway | 92 | 3251 | - | - | - | 16* | 17.5* | Questionnaire | No |
|  |  |  |  |  |  |  |  |  |  |  |  |
| Zarrett et al., 2014 [[69](#_ENREF_69)] | MADICS | USA | 93 | 1037 | 49 | 66% African 30% white | - | 12.8 (2.0) | 16.5 | Questionnaire | Yes |

Date of baseline data collection: last two digits of year e.g. 00 represents 2000

*estimated from other data in the paper

^#^ only at baseline

~different physical activity measure at baseline and follow-up

AT: active transport

SP: leisure time sports participation

+only boys eligible based on age groupings

NR: not reported

Supplementary Table 4: Risk of bias assessment scores

| **Paper** | **Rate selection** | **Rate N** | **Rate drop out** | **Rate tool** | **Rate analyses** | **Overall rating** |
| --- | --- | --- | --- | --- | --- | --- |
| Adachi et al., 2014 [[1](#_ENREF_1)] | Moderate | Strong | Moderate | Weak | Weak | Weak |
| Adachi et al., 2016 [[2](#_ENREF_2)] | Moderate | Strong | Moderate | Weak | Weak | Weak |
| Aires et al., 2012 [[3](#_ENREF_3)] | Weak | Moderate | Weak | Weak | Weak | Weak |
| Andersen et al., 1993 [[4](#_ENREF_4)] | Moderate | Moderate | Strong | Weak | Moderate | Weak |
| Andersen et al., 1994 [[5](#_ENREF_5)] | Moderate | Moderate | Strong | Weak | Moderate | Weak |
| Audrain-McGovern et al., 2012 [[6](#_ENREF_6)] | Moderate | Strong | Strong | Moderate | Moderate | Moderate |
| Bagur-Calafat et al., 2015 [[7](#_ENREF_7)] | Weak | Weak | Strong | Weak | Moderate | Weak |
| Barnett et al., 2013 [[8](#_ENREF_8)] | Weak | Strong | Strong | Weak | Strong | Weak |
| Baxter-Jones et al., 2008 [[9](#_ENREF_9)] | Weak | Moderate | Weak | Moderate | Weak | Weak |
| Benitez-Porres et al., 2016 [[10](#_ENREF_10)] | Weak | Moderate | Moderate | Moderate | Weak | Weak |
| Birkeland et al., 2009 [[11](#_ENREF_11)] | Moderate | Moderate | Moderate | Weak | Weak | Weak |
| Boone-Heinonen et al., 2010 [[12](#_ENREF_12)] | Moderate | Strong | Moderate | Weak | Weak | Weak |
| Boreham et al., 2004 [[13](#_ENREF_13)] | Weak | Moderate | Weak | Weak | Weak | Weak |
| Campbell et al., 2001 [[14](#_ENREF_14)] | Weak | Weak | Weak | Moderate | Weak | Weak |
| Collings et al., 2015 [[15](#_ENREF_15)] | Weak | Moderate | Weak | Strong | Strong | Weak |
| Crocker et al., 2003 [[16](#_ENREF_16)] | Weak | Moderate | Strong | Moderate | Moderate | Weak |
| de Souza et al., 2015 [[17](#_ENREF_17)] | Weak | Moderate | Weak | Moderate | Weak | Weak |
| Deforche et al., 2015 [[18](#_ENREF_18)] | Weak | Moderate | Weak | Moderate | Moderate | Weak |
| Deheeger et al., 2002 [[19](#_ENREF_19)] | Weak | Weak | Weak | Weak | Weak | Weak |
| Eime et al., 2016 [[20](#_ENREF_20)] | Weak | Moderate | Moderate | Weak | Weak | Weak |
| Fortier et al., 2001 [[21](#_ENREF_21)] | Weak | Weak | Weak | Moderate | Weak | Weak |
| Freitas et al., 2012 [[22](#_ENREF_22)] | Weak | Moderate | Weak | Moderate | Weak | Weak |
| Gordon-Larsen et al., 2001 [[23](#_ENREF_23)] | Moderate | Strong | Moderate | Weak | Weak | Weak |
| Graham et al., 2011 [[24](#_ENREF_24)] | Weak | Strong | Weak | Weak | Weak | Weak |
| Gunnell et al., (2016) [[25](#_ENREF_25)] | Weak | Weak | Moderate | Strong | Weak | Weak |
| Han et al., 2008 [[26](#_ENREF_26)] | Weak | Weak | Moderate | Moderate | Moderate | Weak |
| Hasselstrom et al., 2002 [[27](#_ENREF_27)] | Strong | Moderate | Moderate | Weak | Weak | Weak |
| Hearst et al., 2012 [[28](#_ENREF_28)] | Weak | Strong | Strong | Strong | Strong | Strong |
| Hobin et al., 2014 [[29](#_ENREF_29)] | Weak | Moderate | Moderate | Moderate | Moderate | Weak |
| Hunter et al., 2016 [[30](#_ENREF_30)] | Moderate | Weak | Moderate | Weak | Weak | Moderate |
| Huppertz et al., 2016 [[31](#_ENREF_31)] | Weak | Weak | Weak | Weak | Weak | Weak |
| Janssens et al., 2014 [[32](#_ENREF_32)] | Moderate | Strong | Moderate | Weak | Weak | Weak |
| Jung et al., 2008 [[33](#_ENREF_33)] | Weak | Moderate | Moderate | Weak | Moderate | Weak |
| Kahn et al., 2008 [[34](#_ENREF_34)] | Moderate | Moderate | Weak | Moderate | Strong | Weak |
| Kayihan et al., 2014 [[35](#_ENREF_35)] | Weak | Weak | Weak | Moderate | Moderate | Weak |
| Kimm et al., 2002 [[36](#_ENREF_36)] | Moderate | Strong | Strong | Weak | Strong | Weak |
| Kwon et al., 2015 [[37](#_ENREF_37)] | Weak | Moderate | Weak | Strong | Strong | Weak |
| Kwon et al., 2015 [[38](#_ENREF_38)] | Moderate | Strong | Strong | Moderate | Weak | Weak |
| Lantz et al., 2008 [[39](#_ENREF_39)] | Weak | Weak | Weak | Weak | Weak | Weak |
| Lappe et al., 2014 [[40](#_ENREF_40)] | Weak | Strong | Weak | Weak | Weak | Weak |
| Lemoyne et al., 2016 [[41](#_ENREF_41)] | Weak | Weak | Weak | Weak | Weak | Weak |
| Magarey et al., 1999 [[42](#_ENREF_42)] | Weak | Moderate | Weak | Weak | Weak | Weak |
| Martin et al., 2010 [[43](#_ENREF_43)] | Weak | Moderate | Weak | Moderate | Weak | Weak |
| Mitchell et al., 2012 [[44](#_ENREF_44)] | Weak | Strong | Weak | Strong | Weak | Weak |
| Nigg 2001 [[45](#_ENREF_45)] | Weak | Moderate | Weak | Moderate | Moderate | Weak |
| Nordstrom et al., 2008 [[46](#_ENREF_46)] | Weak | Weak | Weak | Weak | Weak | Weak |
| Ortega et al., 2013 [[47](#_ENREF_47)] | Weak | Moderate | Weak | Strong | Strong | Weak |
| Palakshappa et al., 2015 [[48](#_ENREF_48)] | Weak | Strong | Weak | Weak | Strong | Weak |
| Pinto et al., 1998 [[49](#_ENREF_49)] | Weak | Moderate | Moderate | Weak | Weak | Weak |
| Porkka et al., 1997 [[50](#_ENREF_50)] | Moderate | Moderate | Weak | Weak | Moderate | Weak |
| Ramires et al., 2016 [[51](#_ENREF_51)] | Weak | Strong | Weak | Weak | Weak | Weak |
| Rauner et al., 2015 [[52](#_ENREF_52)] | Moderate | Moderate | Weak | Moderate | Moderate | Weak |
| Raustorp et al., 2013 [[53](#_ENREF_53)] | Weak | Weak | Weak | Strong | Moderate | Weak |
| Richards et al., 2009 [[54](#_ENREF_54)] | Weak | Moderate | Strong | Moderate | Strong | Weak |
| Rockette-Wagner et al., 2016 [[55](#_ENREF_55)] | Strong | Strong | Strong | Strong | Strong | Strong |
| Sagatun et al., 2008 [[56](#_ENREF_56)] | Moderate | Strong | Moderate | Moderate | Strong | Moderate |
| Schipperijn et al., 2015 [[57](#_ENREF_57)] | Weak | Moderate | Weak | Strong | Weak | Weak |
| Shi et al., 2006 [[58](#_ENREF_58)] | Weak | Weak | Moderate | Weak | Weak | Weak |
| Simons et al., 2015 [[59](#_ENREF_59)] | Weak | Moderate | Moderate | Weak | Strong | Weak |
| Small et al., 2012 [[60](#_ENREF_60)] | Moderate | Strong | Strong | Weak | Strong | Weak |
| Stavrakakis et al., 2012 [[61](#_ENREF_61)] | Weak | Strong | Strong | Weak | Weak | Weak |
| Taymoori et al., 2011 [[62](#_ENREF_62)] | Weak | Strong | Moderate | Moderate | Moderate | Weak |
| Telama et al., 2014 [[63](#_ENREF_63)] | Strong | Moderate | Weak | Weak | Weak | Weak |
| Telford et al., 2012 [[64](#_ENREF_64)] | Weak | Moderate | Weak | Strong | Weak | Weak |
| van de Laar et al., 2010 [[65](#_ENREF_65)] | Weak | Moderate | Weak | Weak | Weak | Weak |
| van Dyck et al., 2014 [[66](#_ENREF_66)] | Moderate | Moderate | Weak | Weak | Strong | Weak |
| Wagnsson et al., 2014 [[67](#_ENREF_67)] | Moderate | Moderate | Moderate | Weak | Moderate | Weak |
| Wichstrom et al., 2013 [[68](#_ENREF_68)] | Weak | Strong | Weak | Weak | Weak | Weak |
| Zarrett et al., 2014 [[69](#_ENREF_69)] | Moderate | Strong | Moderate | Weak | Weak | Weak |

**References for:**

Supplementary Tables (Corder et al, BJSM 2017)

1. Adachi PJC, Willoughby T: **It's not how much you play, but how much you enjoy the game: the longitudinal associations between adolescents' self-esteem and the frequency versus enjoyment of involvement in sports**. *Journal Of Youth And Adolescence* 2014, **43**:137-145.

2. Adachi PJC, Willoughby T: **From the couch to the sports field: The longitudinal associations between sports video game play, self-esteem, and involvement in sports**. *Psychology of Popular Media Culture* 2015, **4**:329-341.

3. Aires L, Silva G, Martins C, Santos MP, Ribeiro JC, Mota J: **Influence of activity patterns in fitness during youth**. *International Journal Of Sports Medicine* 2012, **33**:325-329.

4. Andersen LB, Haraldsdottir J: **TRACKING OF CARDIOVASCULAR-DISEASE RISK-FACTORS INCLUDING MAXIMAL OXYGEN-UPTAKE AND PHYSICAL-ACTIVITY FROM LATE TEENAGE TO ADULTHOOD - AN 8-YEAR FOLLOW-UP-STUDY**. *Journal Of Internal Medicine* 1993, **234**:309-315.

5. Andersen LB: **Changes in physical activity are reflected in changes in fitness during late adolescence. A 2-year follow-up study**. *The Journal Of Sports Medicine And Physical Fitness* 1994, **34**:390-397.

6. Audrain-McGovern J, Rodriguez D, Rodgers K, Cuevas J, Sass J: **Longitudinal variation in adolescent physical activity patterns and the emergence of tobacco use**. *J Pediatr Psychol* 2012, **37**:622-633.

7. Bagur-Calafat C, Farrerons-Minguella J, Girabent-Farres M, Serra-Grima JR: **The impact of high level basketball competition, calcium intake, menses, and hormone levels in adolescent bone density: a three-year follow-up**. *J Sports Med Phys Fitness* 2015, **55**:58-67.

8. Barnett TA, Maximova K, Sabiston CM, Van Hulst A, Brunet J, Castonguay AL, Bélanger M, O'Loughlin J: **Physical activity growth curves relate to adiposity in adolescents**. *Ann Epidemiol* 2013, **23**:529-533.

9. Baxter-Jones ADG, Eisenmann JC, Mirwald RL, Faulkner RA, Bailey DA: **The influence of physical activity on lean mass accrual during adolescence: A longitudinal analysis**. *Journal of Applied Physiology* 2008, **105**:734-741.

10. Benitez-Porres J, Alvero-Cruz JR, Carrillo de Albornoz M, Correas-Gomez L, Barrera-Exposito J, Dorado-Guzman M, Moore JB, Carnero EA: **The Influence of 2-Year Changes in Physical Activity, Maturation, and Nutrition on Adiposity in Adolescent Youth**. *PloS one* 2016, **11**(9):e0162395.

11. Birkeland MS, Torsheim T, Wold B: **A longitudinal study of the relationship between leisure-time physical activity and depressed mood among adolescents**. *Psychol Sport Exerc* 2009, **10**:25-34.

12. Boone-Heinonen J, Guilkey DK, Evenson KR, Gordon-Larsen P: **Residential self-selection bias in the estimation of built environment effects on physical activity between adolescence and young adulthood**. *Int J Behav Nutr Phys Act* 2010, **7**:70.

13. Boreham C, Robson PJ, Gallagher AM, Cran GW, Savage M, Murray LJ: **Tracking of physical activity, fitness, body composition and diet from adolescence to young adulthood: The young hearts project, Northern Ireland**. *International Journal of Behavioral Nutrition and Physical Activity* 2004, **1**:no pagination.

14. Campbell PT, Katzmarzyk PT, Malina RM, Rao DC, Perusse L, Bouchard C: **Prediction of physical activity and physical work capacity (PWC150) in young adulthood from childhood and adolescence with consideration of parental measures**. *Am J Hum Biol* 2001, **13**:190-196.

15. Collings PJ, Wijndaele K, Corder K, Westgate K, Ridgway CL, Sharp SJ, Dunn V, Goodyer I, Ekelund U, Brage S: **Magnitude and determinants of change in objectively-measured physical activity, sedentary time and sleep duration from ages 15 to 17.5y in UK adolescents: the ROOTS study**. *International Journal of Behavioral Nutrition and Physical Activity* 2015, **12**:10.

16. Crocker P, Sabiston C, Forrestor S, Kowalski N, Kowalski K, McDonough M: **Predicting change in physical activity, dietary restraint, and physique anxiety in adolescent girls: examining covariance in physical self-perceptions**. *Can J Public Health* 2003, **94**:332-337.

17. De Souza MC, Eisenmann JC, D.V ES, De Chaves RN, De Moraes Forjaz CL, Maia JAR: **Modeling the dynamics of BMI changes during adolescence. the oporto growth, health and performance study**. *Int J Obes* 2015, **39**:1063-1069.

18. Deforche B, Van Dyck D, Deliens T, De Bourdeaudhuij I: **Changes in weight, physical activity, sedentary behaviour and dietary intake during the transition to higher education: a prospective study**. *Int J Behav Nutr Phys Act* 2015, **12**:16.

19. Deheeger M, Bellisle F, Rolland-Cachera MF: **The French longitudinal study of growth and nutrition: data in adolescent males and females**. *J Hum Nutr Diet* 2002, **15**:429-438.

20. Eime RM, Harvey JT, Sawyer NA, Craike MJ, Symons CM, Payne WR: **Changes in sport and physical activity participation for adolescent females: a longitudinal study**. *BMC Public Health* 2016, **16**:533.

21. Fortier MD, Katzmarzyk PT, Malina RM, Bouchard C: **Seven-year stability of physical activity and musculoskeletal fitness in the Canadian population**. *Med Sci Sports Exerc* 2001, **33**:1905-1911.

22. Freitas D, Beunen G, Maia J, Claessens A, Thomis M, Marques A, Gouveia É, Lefevre J: **Tracking of fatness during childhood, adolescence and young adulthood: a 7-year follow-up study in Madeira Island, Portugal**. *Ann Hum Biol* 2012, **39**:59-67.

23. Gordon-Larsen P, Adair LS, Popkin BM: **Ethnic differences in physical activity and inactivity patterns and overweight status**. *Obes Res* 2002, **10**:141-149.

24. Graham DJ, Sirard JR, Neumark-Sztainer D: **Adolescents' attitudes toward sports, exercise, and fitness predict physical activity 5 and 10 years later**. *Prev Med* 2011, **52**:130-132.

25. Gunnell KE, Flament MF, Buchholz A, Henderson KA, Obeid N, Schubert N, Goldfield GS: **Examining the bidirectional relationship between physical activity, screen time, and symptoms of anxiety and depression over time during adolescence**. *Prev Med* 2016, **88**:147-152.

26. Han JL, Dinger MK, Hull HR, Randall NB, Heesch KC, Fields DA: **Changes in women's physical activity during the transition to college**. *American Journal of Health Education* 2008, **39**:194-199.

27. Hasselstrøm H, Hansen SE, Froberg K, Andersen LB, Hasselstrom H, Hansen SE, Froberg K, Andersen LB: **Physical fitness and physical activity during adolescence as predictors of cardiovascular disease risk in young adulthood. Danish Youth and Sports Study. An eight-year follow-up study**. *Int J Sports Med* 2002, **23 Suppl 1**:S27-S31.

28. Hearst MO, Patnode CD, Sirard JR, Farbakhsh K, Lytle LA: **Multilevel predictors of adolescent physical activity: a longitudinal analysis**. *Int J Behav Nutr Phys Act* 2012, **9**:8.

29. Hobin E, So J, Rosella L, Comte M, Manske S, McGavock J: **Trajectories of objectively measured physical activity among secondary students in Canada in the context of a province-wide physical education policy: a longitudinal analysis**. *J Obes* 2014, **2014**:958645.

30. Hunter S, Leatherdale ST, Storey K, Carson V: **A quasi-experimental examination of how school-based physical activity changes impact secondary school student moderate- to vigorous- intensity physical activity over time in the COMPASS study**. *Int J Behav Nutr Phys Act* 2016, **13**:86.

31. Huppertz C, Bartels M, de Geus EJC, van Beijsterveldt CEM, Rose RJ, Kaprio J, Silventoinen K: **The effects of parental education on exercise behavior in childhood and youth: A study in Dutch and Finnish twins**. *Scandinavian Journal of Medicine and Science in Sports* 2016.

32. Janssens KAM, Oldehinkel AJ, Bonvanie IJ, Rosmalen JGM: **An inactive lifestyle and low physical fitness are associated with functional somatic symptoms in adolescents. The TRAILS study**. *J Psychosom Res* 2014, **76**:454-457.

33. Jung ME, Bray SR, Ginis KAM: **Behavior Change and the Freshman 15: Tracking Physical Activity and Dietary Patterns in 1st-Year University Women**. *Journal of American College Health* 2008, **56**:523-530.

34. Kahn JA, Huang B, Gillman MW, Field AE, Austin SB, Colditz GA, Frazier AL: **Patterns and determinants of physical activity in U.S. adolescents**. *J Adolesc Health* 2008, **42**:369-377.

35. Kayihan G: **Effect of physical activity on body composition changes in young adults. A four-year longitudinal study**. *Medicina dello Sport* 2014, **67**:423-435.

36. Kimm SY, Glynn NW, Kriska AM, Barton BA, Kronsberg SS, Daniels SR, Crawford PB, Sabry ZI, Liu K: **Decline in physical activity in black girls and white girls during adolescence**. *N Engl J Med* 2002, **347**:709-715.

37. Kwon S, Lee J, Carnethon MR: **Developmental trajectories of physical activity and television viewing during adolescence among girls: National Growth and Health Cohort Study**. *BMC Public Health* 2015, **15**:667.

38. Kwon S, Janz KF, Letuchy EM, Burns TL, Levy SM: **Developmental Trajectories of Physical Activity, Sports, and Television Viewing During Childhood to Young Adulthood: Iowa Bone Development Study**. *JAMA Pediatr* 2015, **169**:666-672.

39. Lantz H, Bratteby LE, Fors H, Sandhagen B, Sjöström L, Samuelson G: **Body composition in a cohort of Swedish adolescents aged 15, 17 and 20.5 years**. *Acta Paediatrica, International Journal of Paediatrics* 2008, **97**:1691-1697.

40. Lappe JM, Watson P, Gilsanz V, Hangartner T, Kalkwarf HJ, Oberfield S, Shepherd J, Winer KK, Zemel B: **The longitudinal effects of physical activity and dietary calcium on bone mass accrual across stages of pubertal development**. *J Bone Miner Res* 2015, **30**:156-164.

41. Lemoyne J, Valois P, Wittman W: **Analyzing Exercise Behaviors during the College Years: Results from Latent Growth Curve Analysis**. *PloS one* 2016, **11**(4):e0154377.

42. Magarey AM, Boulton TJ, Chatterton BE, Schultz C, Nordin BE: **Familial and environmental influences on bone growth from 11-17 years**. *Acta Paediatr* 1999, **88**:1204-1210.

43. Martin AJ, Liem GAD, Coffey L, Martinez C, Parker PP, Marsh HW, Jackson SA: **What happens to physical activity behavior, motivation, self-concept, and flow after completing school? a longitudinal study**. *Journal of Applied Sport Psychology* 2010, **22**:437-457.

44. Mitchell JA, Pate RR, Dowda M, Mattocks C, Riddoch C, Ness AR, Blair SN: **A prospective study of sedentary behavior in a large cohort of youth**. *Med Sci Sports Exerc* 2012, **44**:1081-1087.

45. Nigg CR: **Explaining adolescent exercise behavior change: a longitudinal application of the transtheoretical model**. *Ann Behav Med* 2001, **23**:11-20.

46. Nordstrom A, Neovius MG, Rossner S, Nordstrom P: **Postpubertal development of total and abdominal percentage body fat: An 8-year longitudinal study**. *Obesity* 2008, **16**:2342-2347.

47. Ortega FB, Konstabel K, Pasquali E, Ruiz JR, Hurtig-Wennlof A, Maestu J, Lof M, Harro J, Bellocco R, Labayen I *et al*: **Objectively measured physical activity and sedentary time during childhood, adolescence and young adulthood: a cohort study**. *PloS one* 2013, **8**:e60871.

48. Palakshappa D, Virudachalam S, Oreskovic NM, Goodman E: **Adolescent Physical Education Class Participation as a Predictor for Adult Physical Activity**. *Child Obes* 2015, **11**:616-623.

49. Pinto BM, Cherico NP, Szymanski L, Marcus BH: **Longitudinal changes in college students' exercise participation**. *J Am Coll Health* 1998, **47**:23-27.

50. Porkka KV, Raitakari OT, Leino A, Laitinen S, Rasanen L, Ronnemaa T, Marniemi J, Lehtimaki T, Taimela S, Dahl M *et al*: **Trends in serum lipid levels during 1980-1992 in children and young adults. The Cardiovascular Risk in Young Finns Study**. *Am J Epidemiol* 1997, **146**:64-77.

51. Ramires VV, Dumith SC, Wehrmeister FC, Hallal PC, Menezes AM, Goncalves H: **Physical activity throughout adolescence and body composition at 18 years: 1993 Pelotas (Brazil) birth cohort study**. *Int J Behav Nutr Phys Act* 2016, **13**(1):105.

52. Rauner A, Jekauc D, Mess F, Schmidt S, Woll A: **Tracking physical activity in different settings from late childhood to early adulthood in Germany: The MoMo longitudinal study Health behavior, health promotion and society**. *BMC Public Health* 2015, **15**:391.

53. Raustorp A, Ekroth Y: **Tracking of pedometer-determined physical activity: a 10-year follow-up study from adolescence to adulthood in Sweden**. *Journal of physical activity & health* 2013, **10**:1186-1192.

54. Richards R, Poulton R, Reeder AI, Williams S: **Childhood and contemporaneous correlates of adolescent leisure time physical inactivity: a longitudinal study**. *J Adolesc Health* 2009, **44**:260-267.

55. Rockette-Wagner B, Hipwell AE, Kriska AM, Storti KL, McTigue KM: **Activity Levels over Four Years in a Cohort of Urban-Dwelling Adolescent Females**. *Med Sci Sports Exerc* 2016.

56. Sagatun Å, Kolle E, Anderssen SA, Thoresen M, Søgaard AJ: **Three-year follow-up of physical activity in Norwegian youth from two ethnic groups: Associations with socio-demographic factors**. *BMC Public Health* 2008, **8**.

57. Schipperijn J, Ried-Larsen M, Nielsen MS, Holdt AF, Grøntved A, Ersbøll AK, Kristensen PL: **A longitudinal study of objectively measured built environment as determinant of physical activity in young adults: The European Youth Heart Study**. *Journal of Physical Activity and Health* 2015, **12**:909-914.

58. Shi HJ, Nakamura K, Kizuki M, Inose T, Seino K, Takano T: **Extracurricular sports activity around growth spurt and improved tibial cortical bone properties in late adolescence**. *Acta Paediatr* 2006, **95**:1608-1613.

59. Simons D, Rosenberg M, Salmon J, Knuiman M, Granich J, Deforche B, Timperio A: **Psychosocial moderators of associations between life events and changes in physical activity after leaving high school**. *Prev Med* 2015, **72**:30-33.

60. Small M, Bailey-Davis L, Morgan N, Maggs J: **Changes in eating and physical activity behaviors across seven semesters of college: living on or off campus matters**. *Health Educ Behav* 2013, **40**:435-441.

61. Stavrakakis N, de Jonge P, Ormel J, Oldehinkel AJ: **Bidirectional prospective associations between physical activity and depressive symptoms. The TRAILS Study**. *J Adolesc Health* 2012, **50**:503-508.

62. Taymoori P, Berry TR, Lubans DR: **Tracking of physical activity during middle school transition in Iranian adolescents**. *Health Educ J* 2012, **71**:631-641.

63. Telama R, Yang X, Leskinen E, Kankaanpää A, Hirvensalo M, Tammelin T, Viikari JSA, Raitakari OT: **Tracking of physical activity from early childhood through youth into adulthood**. *Med Sci Sports Exerc* 2014, **46**:955-962.

64. Telford A, Finch CF, Barnett L, Abbott G, Salmon J: **Do parents' and children's concerns about sports safety and injury risk relate to how much physical activity children do?** *Br J Sports Med* 2012, **46**:1084-1088.

65. Van De Laar RJ, Ferreira I, Van Mechelen W, Prins MH, Twisk JW, Stehouwer CD: **Lifetime vigorous but not light-to-moderate habitual physical activity impacts favorably on carotid stiffness in young adults: The amsterdam growth and health longitudinal study**. *Hypertension* 2010, **55**:33-39.

66. Van Dyck D, De Bourdeaudhuij I, Deliens T, Deforche B, Dyck D, Bourdeaudhuij I, Deliens T, Deforche B: **Can changes in psychosocial factors and residency explain the decrease in physical activity during the transition from high school to college or university?** *Int J Behav Med* 2015, **22**:178-186.

67. Wagnsson S, Lindwall M, Gustafsson H: **Participation in organized sport and self-esteem across adolescence: the mediating role of perceived sport competence**. *J Sport Exerc Psychol* 2014, **36**:584-594.

68. Wichstrom L, von Soest T, Kvalem IL: **Predictors of growth and decline in leisure time physical activity from adolescence to adulthood**. *Health Psychol* 2013, **32**:775-784.

69. Zarrett N, Bell BA: **The effects of out-of-school time on changes in youth risk of obesity across the adolescent years**. *J Adolesc* 2014, **37**:85-96.
