# Supplementary material for: Change in physical activity from adolescence to early adulthood: a systematic review and meta-analysis of longitudinal cohort studies
Source: Br J Sports Med. 2017 Jul 24;53(8):496–503. doi: 10.1136/bjsports-2016-097330 (PMC6250429; doi:10.1136/bjsports-2016-097330)
Supplement: Supplementary file 2 [file bjsports-2016-097330supp002.pdf]

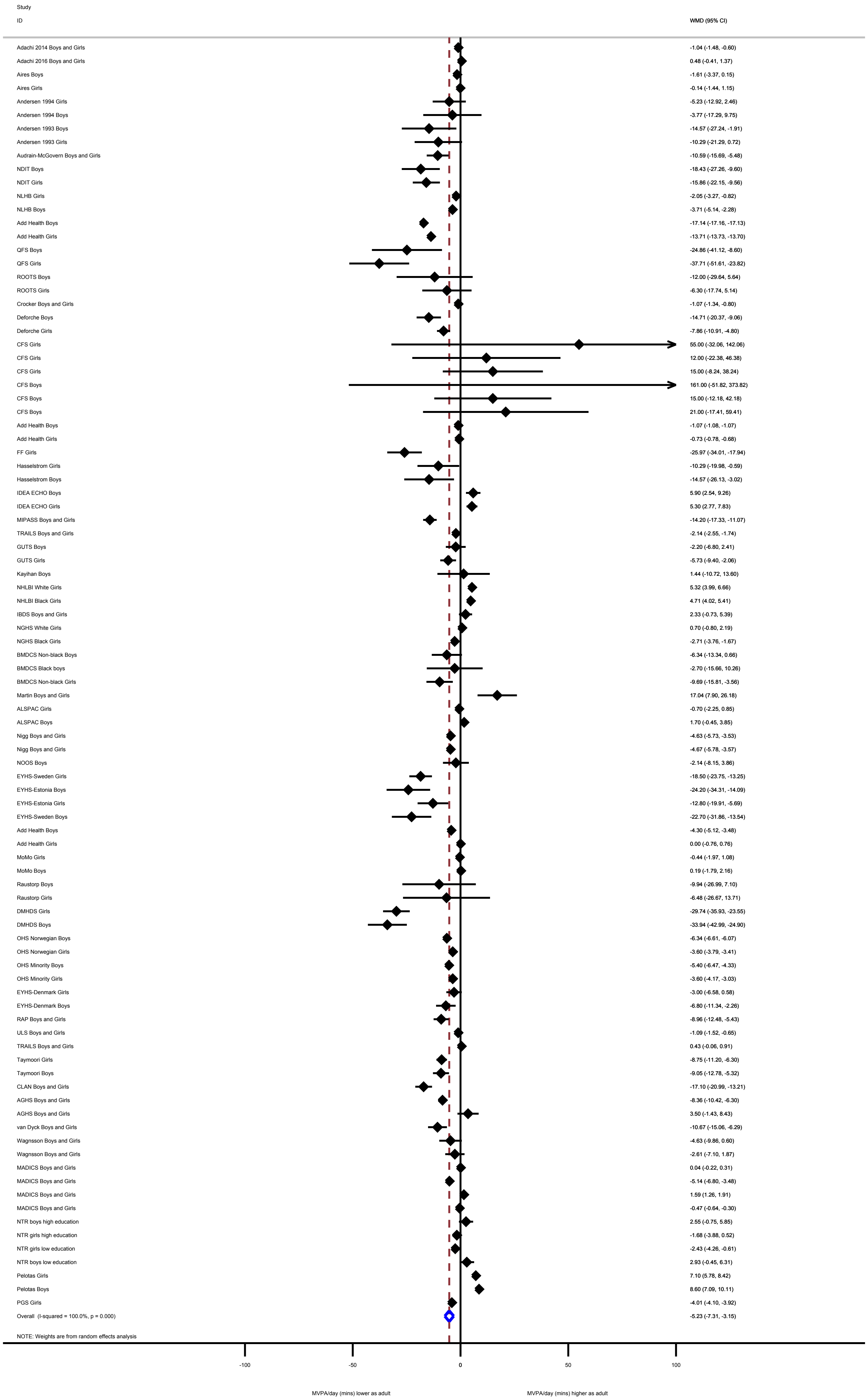
Study
ID
WMD (95% CI)
Adachi 2014 Boys and Girls -1.04 (-1.48, -0.60)
Adachi 2016 Boys and Girls 0.48 (-0.41, 1.37)
Aires Boys -1.61 (-3.37, 0.15)
Aires Girls -0.14 (-1.44, 1.15)
Andersen 1994 Girls -5.23 (-12.92, 2.46)
Andersen 1994 Boys -3.77 (-17.29, 9.75)
Andersen 1993 Boys -14.57 (-27.24, -1.91)
Andersen 1993 Girls -10.29 (-21.29, 0.72)
Audrain-McGovern Boys and Girls -10.59 (-15.69, -5.48)
NDIT Boys -18.43 (-27.26, -9.60)
NDIT Girls -15.86 (-22.15, -9.56)
NLHB Girls -2.05 (-3.27, -0.82)
NLHB Boys -3.71 (-5.14, -2.28)
Add Health Boys -17.14 (-17.16, -17.13)
Add Health Girls -13.71 (-13.73, -13.70)
QFS Boys -24.86 (-41.12, -8.60)
QFS Girls -37.71 (-51.61, -23.82)
ROOTS Boys -12.00 (-29.64, 5.64)
ROOTS Girls -6.30 (-17.74, 5.14)
Crocker Boys and Girls -1.07 (-1.34, -0.80)
Deforche Boys -14.71 (-20.37, -9.06)
Deforche Girls -7.86 (-10.91, -4.80)
CFS Girls 55.00 (-32.06, 142.06)
CFS Girls 12.00 (-22.38, 46.38)
CFS Girls 15.00 (-8.24, 38.24)
CFS Boys 161.00 (-51.82, 373.82)
CFS Boys 15.00 (-12.18, 42.18)
CFS Boys 21.00 (-17.41, 59.41)
Add Health Boys -1.07 (-1.08, -1.07)
Add Health Girls -0.73 (-0.78, -0.68)
FF Girls -25.97 (-34.01, -17.94)
Hasselstrom Girls -10.29 (-19.98, -0.59)
Hasselstrom Boys -14.57 (-26.13, -3.02)
IDEA ECHO Boys 5.90 (2.54, 9.26)
IDEA ECHO Girls 5.30 (2.77, 7.83)
MIPASS Boys and Girls -14.20 (-17.33, -11.07)
TRAILS Boys and Girls -2.14 (-2.55, -1.74)
GUTS Boys -2.20 (-6.80, 2.41)
GUTS Girls -5.73 (-9.40, -2.06)
Kayihan Boys 1.44 (-10.72, 13.60)
NHLBI White Girls 5.32 (3.99, 6.66)
NHLBI Black Girls 4.71 (4.02, 5.41)
IBDS Boys and Girls 2.33 (-0.73, 5.39)
NGHS White Girls 0.70 (-0.80, 2.19)
NGHS Black Girls -2.71 (-3.76, -1.67)
BMDCS Non-black Boys -6.34 (-13.34, 0.66)
BMDCS Black boys -2.70 (-15.66, 10.26)
BMDCS Non-black Girls -9.69 (-15.81, -3.56)
Martin Boys and Girls 17.04 (7.90, 26.18)
ALSPAC Girls -0.70 (-2.25, 0.85)
ALSPAC Boys 1.70 (-0.45, 3.85)
Nigg Boys and Girls -4.63 (-5.73, -3.53)
Nigg Boys and Girls -4.67 (-5.78, -3.57)
NOOS Boys -2.14 (-8.15, 3.86)
EYHS-Sweden Girls -18.50 (-23.75, -13.25)
EYHS-Estonia Boys -24.20 (-34.31, -14.09)
EYHS-Estonia Girls -12.80 (-19.91, -5.69)
EYHS-Sweden Boys -22.70 (-31.86, -13.54)
Add Health Boys -4.30 (-5.12, -3.48)
Add Health Girls 0.00 (-0.76, 0.76)
MoMo Girls -0.44 (-1.97, 1.08)
MoMo Boys 0.19 (-1.79, 2.16)
Raustorp Boys -9.94 (-26.99, 7.10)
Raustorp Girls -6.48 (-26.67, 13.71)
DMHDS Girls -29.74 (-35.93, -23.55)
DMHDS Boys -33.94 (-42.99, -24.90)
OHS Norwegian Boys -6.34 (-6.61, -6.07)
OHS Norwegian Girls -3.60 (-3.79, -3.41)
OHS Minority Boys -5.40 (-6.47, -4.33)
OHS Minority Girls -3.60 (-4.17, -3.03)
EYHS-Denmark Girls -3.00 (-6.58, 0.58)
EYHS-Denmark Boys -6.80 (-11.34, -2.26)
RAP Boys and Girls -8.96 (-12.48, -5.43)
ULS Boys and Girls -1.09 (-1.52, -0.65)
TRAILS Boys and Girls 0.43 (-0.06, 0.91)
Taymoori Girls -8.75 (-11.20, -6.30)
Taymoori Boys -9.05 (-12.78, -5.32)
CLAN Boys and Girls -17.10 (-20.99, -13.21)
AGHS Boys and Girls -8.36 (-10.42, -6.30)
AGHS Boys and Girls 3.50 (-1.43, 8.43)
van Dyck Boys and Girls -10.67 (-15.06, -6.29)
Wagnsson Boys and Girls -4.63 (-9.86, 0.60)
Wagnsson Boys and Girls -2.61 (-7.10, 1.87)
MADICS Boys and Girls 0.04 (-0.22, 0.31)
MADICS Boys and Girls -5.14 (-6.80, -3.48)
MADICS Boys and Girls 1.59 (1.26, 1.91)
MADICS Boys and Girls -0.47 (-0.64, -0.30)
NTR boys high education 2.55 (-0.75, 5.85)
NTR girls high education -1.68 (-3.88, 0.52)
NTR girls low education -2.43 (-4.26, -0.61)
NTR boys low education 2.93 (-0.45, 6.31)
Pelotas Girls 7.10 (5.78, 8.42)
Pelotas Boys 8.60 (7.09, 10.11)
PGS Girls -4.01 (-4.10, -3.92)
Overall (I-squared = 100.0%, p = 0.000) -5.23 (-7.31, -3.15)
NOTE: Weights are from random effects analysis
-100
-50
0
50
100
MVPA/day (mins) lower as adult
MVPA/day (mins) higher as adult
